# Zoledronic acid alters hematopoiesis and generates breast tumor-suppressive bone marrow cells

**DOI:** 10.1186/s13058-017-0815-8

**Published:** 2017-03-06

**Authors:** Jessalyn M. Ubellacker, Marie-Therese Haider, Molly J. DeCristo, Gloria Allocca, Nicola J. Brown, Daniel P. Silver, Ingunn Holen, Sandra S. McAllister

**Affiliations:** 1 Department of Medicine, Harvard Medical School, Boston, MA 02115 USA; 2 Hematology Division, Brigham & Women’s Hospital, Boston, MA 02115 USA; 3 Department of Oncology & Metabolism, University of Sheffield, Sheffield, UK; 4 Departments of Medical Oncology and Cancer Biology, Sidney Kimmel Cancer Center, Thomas Jefferson University, Philadelphia, PA 19107 USA; 5 Broad Institute of Harvard and MIT, Cambridge, MA 02142 USA; 6 Harvard Stem Cell Institute, Cambridge, MA 02138 USA

**Keywords:** Zoledronic acid, Bisphosphonate, Tumor-suppressive bone marrow cells, Hematopoietic stem cells, Hematopoietic stem cell niches, Hematopoiesis

## Abstract

**Background:**

The bone-targeting agent zoledronic acid (ZOL) increases breast cancer survival in subsets of patients, but the underlying reasons for this protective effect are unknown. ZOL modulates the activity of osteoclasts and osteoblasts, which form hematopoietic stem cell niches, and therefore may affect hematopoietic cells that play a role in breast cancer progression.

**Method:**

Immunocompetent and immunocompromised strains of mice commonly used for breast cancer research were injected with a single, clinically relevant dose of ZOL (100 μg/kg) or vehicle control. The effects of ZOL on the bone marrow microenvironment (bone volume, bone cell number/activity, extracellular matrix composition) were established at various time points following treatment, using micro-computed tomography (μCT) analysis, histomorphometry, ELISA and immunofluorescence. The effects on peripheral blood and bone marrow hematopoietic progenitor populations were assessed using a HEMAVET® hematology analyzer and multicolor flow cytometry, respectively. Tumor support function of bone marrow cells was determined using an in vivo functional assay developed in our laboratory.

**Results:**

Using multiple mouse strains, we observed transient changes in numbers of hematopoietic stem cells, myeloid-biased progenitor cells, and lymphoid-biased cells concurrent with changes to hematopoietic stem cell niches following ZOL administration. Importantly, bone marrow cells from mice treated with a single, clinically relevant dose of ZOL inhibited breast tumor outgrowth in vivo. The ZOL-induced tumor suppressive function of the bone marrow persisted beyond the time point at which numbers of hematopoietic progenitor cells had returned to baseline.

**Conclusions:**

These findings provide novel evidence that alterations to the bone marrow play a role in the anti-tumor activity of ZOL and suggest possibilities for capitalizing on the beneficial effects of ZOL in reducing breast cancer development and progression.

**Electronic supplementary material:**

The online version of this article (doi:10.1186/s13058-017-0815-8) contains supplementary material, which is available to authorized users.

## Background

Breast cancer patients who have bone metastases experience significant morbidity due to the osteolytic nature of the disease [1]. Bisphosphonates inhibit osteoclast activity and are used to treat patients with bone metastases and/or osteoporosis. Recent clinical results showed that women treated with bisphosphonates, including zoledronic acid (ZOL), are at reduced risk of breast cancer development and recurrence [2, 3]. Nevertheless, how ZOL exerts its protective effect is not fully understood.

Breast cancer development and progression involves not only dissemination of tumor cells to the bone but also the recruitment of tumor-supportive hematopoietic progenitor cells from the bone marrow [4], thus opening the possibility of targeting these cell populations as part of anti-cancer therapy. It is therefore important to establish how bone-targeting agents, widely used to treat osteoporosis and breast cancer-induced bone disease, modify progenitor cell populations in the bone marrow.

Within bone, specialized cellular niches sustain hematopoiesis by providing molecular cues that regulate hematopoietic stem cell (HSC) quiescence, proliferation, differentiation and mobilization [5–7]. Under normal physiological conditions, quiescent HSCs reside in less perfused endosteal niches and in greater proximity to osteoblastic cells than actively proliferating HSCs, which are commonly juxtaposed with sinusoidal endothelium and endothelial cells in vascular niches [8, 9]. Given that endosteal and vascular niche components are critical for maintaining hematopoietic homeostasis, it is not surprising that modulation of either of these niches impacts hematopoietic cells, particularly HSCs [10–12]. For example, it is well-established that an elevation in osteoblast numbers results in an increase in HSCs within the bone marrow [10, 13], whereas depletion of cells with osteoblastic differentiation potential decreases the numbers of HSCs [14].

The nitrogen-containing bisphosphonate, ZOL, inhibits enzymes in the mevalonate pathway, thus inducing osteoclast apoptosis and resulting in inhibition of bone resorption [15, 16]. Various in vitro and in vivo studies have identified osteoclasts and monocyte/macrophages as the primary targets of bisphosphonates, due to their high endocytic capacity [16–20]. Emerging evidence supports the theory that ZOL targets additional cell types in bone, including osteoblasts [21] and vascular endothelial cells [22], although whether these effects are direct or indirect, and their impact on hematopoiesis and disseminated tumor cells, remains unclear.

Here we report that a clinically relevant dose of ZOL has significant and transient effects on hematopoiesis in the bone marrow in vivo, concomitant with changes to the endosteal and vascular niches. Importantly, we demonstrate that a single administration of ZOL generates tumor-suppressive bone marrow cells that are capable of inhibiting breast tumor outgrowth when transplanted into new hosts. Our findings provide novel evidence that ZOL-mediated alterations to the bone marrow may play a role in the anti-tumor activity of ZOL observed in breast cancer clinical trials.

## Methods

### Mice

Experiments were performed in 6 to 7-week-old female NCr-Nu (nude), CrTac:NCr-*Foxn1* ^nu^ mice (Taconic Laboratories, Hudson, NY, USA) and 6 to 7-week-old female C57BL/6 mice (The Jackson Laboratory, Bar Harbor, ME, USA). All procedures were performed in accordance with the regulations of Boston Children’s Hospital Institutional Animal Care and Use Committee (protocol 12-11-2308R).

### Zoledronic acid administration

ZOL [1-hydroxy-2-(1H-imidazole-1-yl)ethylidene-bisphosphonic acid] (Zometa, Novartis Pharmaceuticals, Cambridge, MA, USA) was diluted to a working concentration of 4 mg/mL in 1X Hank’s Balanced Buffer Solution (HBBS, Gibco, 14065-056) and stored at 4 °C until use. A single dose of 100 μL of 1X HBBS or 100 μg/kg ZOL was administered to mice via intraperitoneal (i.p.) injection.

### Bone marrow, spleen and blood preparation

Femora and tibiae were dissected free into 2% FBS in PBS. Bone marrow cells (BMCs) were collected by centrifugation of mouse femora and tibiae at 6000–7000 g for 4 minutes at 4 °C. Cells were then incubated with red blood cell (RBC) lysis solution (BioLegend 420301) for 5 minutes on ice, washed once with BMC buffer, re-suspended in 0.5 mL of sterile BMC buffer, and passed through a 5-mL polystyrene round-bottom tube with a cell-strainer cap (Corning, 352235). One fourth of the spleen was mechanically dissociated with a razor blade, incubated with RBC lysis solution (BioLegend 420301) for 5 minutes on ice, washed once with PBS, and filtered through a 70-μM nylon mesh filter. Next, 200 μL of peripheral blood was incubated with RBC lysis solution (BioLegend 420301) for 10 minutes at room temperature, washed once with PBS passed through a 5-mL polystyrene round-bottom tube with a cell-strainer cap (Corning, 352235). At the experimental end points, blood was collected by intracardiac puncture with a 27-gauge needle into ethylenediaminetetraacetic acid (EDTA) Microtainer tubes (BD Pharmingen). Complete blood counts were obtained using a HEMAVET® hematology analyzer (Drew Scientific). Plasma was prepared by centrifugation of whole blood at 1500 g for 8 minutes at 4 °C.

### Flow cytometry

BMCs (preparation as previously described) were incubated with appropriate antibodies for 30 minutes at 4 °C. Dead cells were eliminated from analysis using 7-aminoactinomycin D (7-AAD; BioLegend), and flow cytometry gating was used to exclude debris and cell clumps. Countbright Absolute counting beads (Life Technologies, C36950) were added to the samples to quantify the numbers of cells of any given lineage and report absolute differences between cohorts. Data were acquired on a Canto II or a FACSAria IIu/FACSDiva (BD Biosciences). Data analyses were performed using FlowJo software (TreeStar). Antibody information, dilutions, and cell gating strategies are included in Additional file 1: Table S1, Additional file 2: Table S2 and Additional file 3: Figure S1.

### Bromodeoxyuridine (BrdU) incorporation

BrdU incorporation was performed per manufacturer’s instructions using the BD Pharmigen APC BrdU Flow Kit (Catalog number 552598). Mice were injected i.p. with 100 μL of 1 mg BrdU solution 2 hours prior to tissue harvest. BMCs (preparation as previously described) and cell-surface antigens were stained using the antibodies indicated in Additional file 1: Table S1. Cells were fixed and permeabilized with BD Cytofix/Cytoperm Buffer and treated with 100 μL of 300 μg/mL DNase to expose incorporated BrdU prior to staining with APC BrdU antibody (BD Biosciences, 557892).

### Cell colony assays

BMCs, spleen cells, and peripheral blood cells (preparation as previously described) were passed through a 70-μM nylon mesh filter. BMCs (3 × 10^4^) were prepared and plated into 35-mm Petri dishes in Mouse Colony-Forming Unit (CFU) Methocult™ Assays (Stemcell Technologies Inc., M3434) according to the manufacturer’s instructions. Colonies were counted after 10 days of culture.

### Ki67 immunohistochemistry and quantification

Femora were dissected free and fixed in 4% (wt/vol) paraformaldehyde (PFA) for 24 hours, stored in 70% ethanol for 24 hours, embedded in paraffin, and sectioned onto ProbeOn Plus slides (Fisher Scientific). Tibiae were dissected free and fixed in ice cold 4% PFA (pH 7.4 in PBS) for 48–72 hours and decalcified in 0.5 M EDTA/0.5% PFA (pH8, in PBS) for 2 weeks at 4 °C followed by paraffin embedding. Proliferating BMCs were identified by staining with an antibody to Ki67 (Abcam, catalog number ab15580, dilution 1:200) and a secondary Vectastain kit (ABC, Rabbit IGG, PK-6101) with AEC Chromogen Substrate (Dako, K3461). Tissues were counter stained with hematoxylin (Vector laboratories, VWR101098-062). Images were captured with identical exposure and gain for any given experiment, using a Nikon Eclipse Ni microscope. Staining was quantified using ImageJ (NIH) and CellProfiler (The Broad Institute) image analysis software.

### Osteoclast (TRAP) and osteoblast quantifications

Osteoblast and osteoclast number/mm trabecular bone was quantified on H&E or tartrate-resistant acid phosphatase (TRAP)-stained sections of tibiae covering all trabecular bone surfaces 200 μm away from the epiphysis, as described previously [21]. Osteoblasts were identified by their large Golgi complex, single, distinct nucleus, cuboidal shape and proximity to endosteal surfaces. Osteoclasts were identified by their bright pink appearance after TRAP staining. Two non-serial 3-μm-thick sections were assessed per sample using an Olympus BX53 (×20 objective) and OsteoMeasure software (Osteometrics). Detailed scoring methodologies are illustrated in Additional file 4: Figure S2A.

### Extracellular matrix deposition

Proteoglycan-rich extracellular matrix in the epiphysis and metaphysis were visualized by toluidine blue staining on sections of tibiae. Two non-serial 3-μm-thick sections were assessed per sample using an Olympus BX53 a Leica DMRB microscope (×2.5 objective) and the area of interest (AOI) within a total tissue area of 1 mm^2^ was calculated using OsteoMeasure software (Osteometrics; see Additional file 4: Figure S2B). Cortical and trabecular bone surfaces not directly connected to the dense network of trabeculi at the epiphysis were excluded from analysis.

### Micro-computed tomography (μCT) analysis

The trabecular bone volume of femora was analyzed using a SkyScan 1272 device (SkyScan). Femora were scanned using 200 mA, 51 kV, a 0.5-mm aluminum filter, medium camera resolution of 2016 × 1344 and pixel size set to 4.3 μm. Images were reconstructed using NRecon software and a bone volume of interest (VOI) was determined by interactively drawing on the two-dimensional images. Analysis was started from a fixed offset 0.7 mm away from the proximal end of the growth plate covering a length of 1.5 mm. Grayscale images of the VOI were converted into binary images (threshold 95 − 225) and bone parameters were calculated using the CTAn software.

### Visualization of bone marrow vasculature

Tibiae were fixed in ice cold 4% PFA (pH 7.4, in PBS) for 4 hours at 4 °C, decalcified in 0.5 M EDTA (pH 8, in PBS) for at least 24 hours, transferred to 20% sucrose/2% polyvinylpyrrolidone (PVP) solution (in PBS) overnight at 4 °C followed by embedding in 8% gelatine/20% sucrose/2% PVP and 30-μm-thick sections were prepared using a cryostat at -20 °C as described by Kusumbe et al. 2014 [23].

Bone marrow vasculature was visualized by immunofluorescent staining using antibodies against the vascular endothelial cell marker Endomucin (1:100, Endomucin V.7C7, rat monoclonal, Santa Cruz, sc-65495) and CD31 (1:100, DIA-310: Anti-CD31 (Ms) from Rat (Clone: SZ31)). Alexa Fluor 555, goat anti-rat IgG, (LifeTechnologies, A21434 (1:200)) was used as the secondary antibody.

Images were acquired using a Nikon A1 confocal microscope, NIS-Elements-software Version 4.30, CFI Plan Fluor 20x MI (NA 0.75) or a Nikon inverted Ti eclipse, NIS-Elements software Version 4.30, Plan Apo 20x (NA 0.75). For a subset of samples, Z-stacks of 20-μm depth (0.9-μm intervals, 23 steps) were acquired and 3D projections reconstructed using Fiji (ImageJ, Version 2.0.0-rc-24/1.49 m, Image > Stacks > 3D projections). Brightness and contrast for the entire images were adjusted using Fiji or NIS-Elements-software. The Nikon Eclipse Ti microscope was used to produce tile scans (8 × 8). Aperio ImageScope software was used to track vessels positively stained for endomucin in an average area of bone marrow of 615 x 1095 μm from the top of the growth plate.

### Cells

Human breast cancer MDA-MB-231 BO2F11 luciferase-transfected bone-tropic cells were a kind gift from the van der Pluijm laboratory (Leiden University Medical Center, The Netherlands) [24]. Cells tested negative for mycoplasma and were validated by short tandem repeat (STR) profiling by the Molecular Diagnostics Laboratory at the Dana-Farber Cancer Institute.

### Bioluminescent imaging

Mice were anesthetized with 2.5% isoflurane and injected i.p. with 150 mg/kg D-luciferin (Perkin-Elmer). Luciferase-positive cells were detected using a Xenogen IVIS imaging system (Caliper Life Sciences) 10 minutes after luciferin injection. Luminescence signal was detected for the regions of interest as radiance (p/sec/cm^2^/sr) and analyzed using the Living Image Software Version 4.1 (Caliper Life Sciences).

### Bone marrow functional analysis and subcutaneous tumor growth

BMCs were freshly harvested from cohorts of donor mice (preparation as described previously) and immediately admixed with MDA-MB-231 BO2F11 tumor cells in DMEM with 10% Matrigel (BD, Growth Factor Reduced, 356230). For each injection, 750,000 BMCs from a given donor mouse were admixed with 250,000 tumor cells in 100 μL DMEM/10% Matrigel. BMCs from each donor mouse were distributed bilaterally into three recipient nude mice, with six possible tumors per donor. Tumor growth was monitored by in vivo imaging system (IVIS) imaging of all mice and caliper measurements were taken for all palpable tumors. Volume was estimated as 0.5 × length × width^2^.

### Statistical analysis

All data were analyzed with the use of GraphPad Prism Software (Version 6). Data are expressed as mean ± SEM. Results were analyzed using Student’s *t* test, unless otherwise indicated, and were considered statistically significant if the *p* value was ≤0.05.

## Results

### Effect of zoledronic acid on hematopoietic stem and progenitor cells

**Fig. 1:**
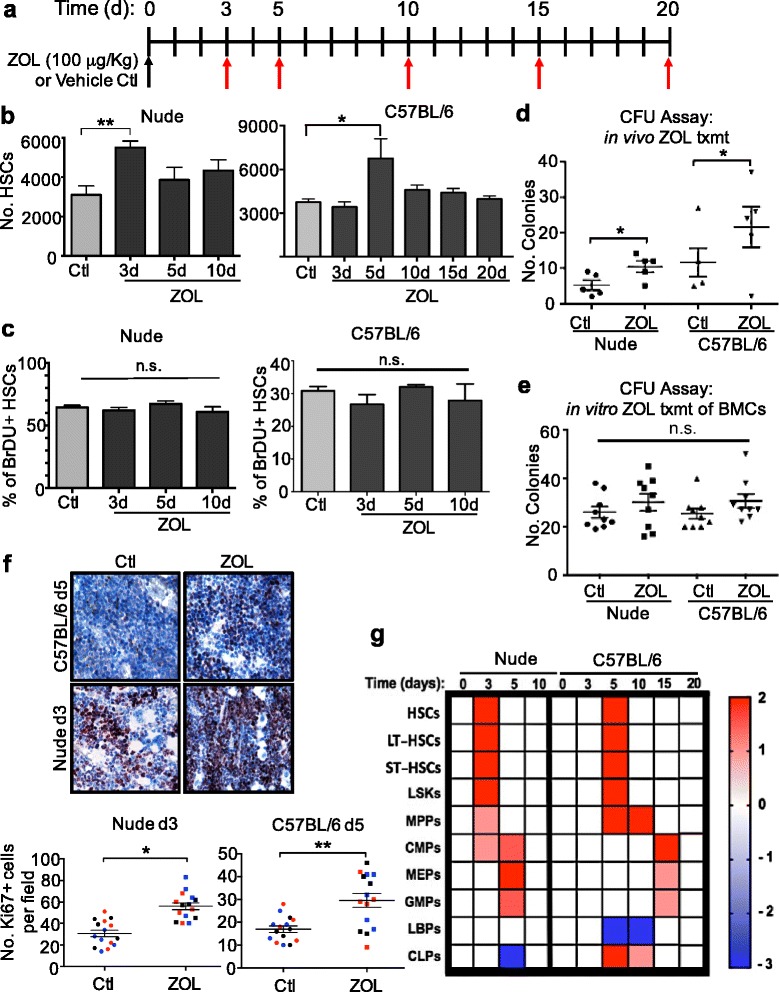
Impact of zoledronic acid (*ZOL*) on hematopoietic and peripheral blood cells. **a** Cohorts of C57BL/6 and nude mice (*n* = 5 per cohort per experiment × three experiments) were administered a single 100-μg/kg dose of ZOL or vehicle control (100 μl Hanks Balanced Salt Solution (HBSS)) via intraperitoneal injection and tissues were analyzed at indicated time points (*red arrows*) (**b**-**d**, **f**, **g**). **b** Number of hematopoietic stem cells (*HSCs*) per nude or C57BL/6 mouse femur at indicated time points after ZOL treatment, determined by flow cytometry using counting beads; **p = 0.003,*p = 0.04 (*n* = 15; 5 mice per cohort for each of three biological replications). **c** Bromodeoxyuridine (*BrDU*)-positive HSCs as a percentage of total HSCs per nude or C57BL/6 mouse femur; *n.s.* not significant; *n* = 10 femora (five mice, two femora per mouse). **d** Colony forming unit (*CFU*) assays using bone marrow cells (*BMCs*) isolated from both vehicle and ZOL-treated mice 3 (nude mice) or 5 days (C57BL/6 mice) after ZOL treatment. Colonies were counted 10 days later; nude mice *p = 0.04, C57BL/6 mice *p = 0.05. **e** BMCs from three naïve C57BL/6 mice and three nude mice were prepared in triplicate and subjected to CFU assay in the presence of 10 μM ZOL or 10 μL of 1X HBBS; colonies were counted after 10 days; *n.s.* not significant. **f** Representative Ki67 immunohistochemical stains of bone marrow from vehicle and ZOL-treated mice 5 days (C57BL/6) or 3 days (nude mice) after treatment (×40 objective). Quantification of Ki67 staining from indicated mice and time points. *Colors* indicate different biological replications; each *data point* represents an individual mouse for which an average of three different fields of view was calculated; nude mice *p < 0.0001, C57BL/6 mice **p = 0.0007. **g** Average fold change in bone marrow hematopoietic progenitor populations (*HPCs*) (quantified from one femur per mouse by flow cytometry; *n* = 5, representative of three biological replications) at indicated time points after one 100 μg/kg ZOL dose as compared to vehicle treatment. *Ctl* control, *LT-HSCs* long-term HSCs, *ST-HSCs* short-term HSCs, *LSKs* ﻿Lin-Sca1+cKit+﻿, *MPPs* multipotent progenitor populations, *CMPs* common myeloid progenitors, *MEPs* megakaryocyte/erythroid progenitors, *GMPs* granulocyte/monocyte progenitors, *LBPs* lymphoid-biased progenitors, *CLPs* common lymphoid progenitors. Also see Table 1

To determine whether ZOL impacts hematopoiesis, we used two different strains of mice - nude and C57BL/6 - that are commonly used in breast cancer research. While patients with osteoporosis or metastatic bone disease are often treated for chronic disease [25], our goal was to evaluate the effects of ZOL on hematopoiesis in the absence of overt bone disease. We also reasoned that effects on hematopoiesis should be analyzed over a time period when ZOL is known to be bioavailable in the bone. ZOL is known to concentrate in the bone within 24 hours of administration and is cleared during bone turnover, which occurs at a rate of around 0.7% per day in the mouse and thus, takes 2 weeks to complete [26]. Hence, we administered a single, clinically relevant dose of 100 μg/kg ZOL (comparable to the 4-mg clinical dose that has been well-established to inhibit osteoclast activity in vivo [21]) to cohorts of immunocompromised (nude) and immunocompetent (C57BL/6) mice and analyzed hematopoietic cells at various time points over a course of 2 weeks (Fig. 1a).

In the nude mice, the numbers of HSCs (LSK/CD150+/CD48-/CD34-/Flt3-) in the marrow of ZOL-treated mice were elevated 1.8-fold (*p* = 0.003) relative to the control cohort 3 days after administration, after which the numbers returned to baseline (Fig. 1b, Table 1). In agreement with these results, numbers of both short-term HSCs (ST-HSCs, LSK Flt3+ CD34-) and long-term HSCs (LT-HSCs, LSK Flt3- CD34-) were elevated 4.1-fold (*p* = 0.01) and 4.8-fold (*p* = 0.06), respectively (Table 1).

**Table 1:**
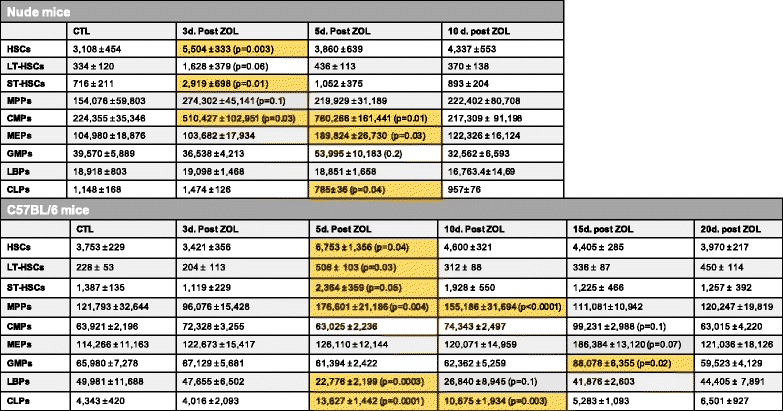
Zoledronic acid-induced modifications to bone marrow hematopoietic and progenitor cell numbers over time

Average numbers of indicated bone marrow HPC populations (number of cells ± SEM) from nude and C57BL/6 mice as determined by flow cytometry on one femur per mouse at indicated time points following control (CTL) or one 100 μg/kg dose of zoledronic acid (ZOL) treatment; *n* = 15; *N* = 5 mice per group × three biological replications. Dark yellow shaded boxes represent statistical significance relative to CTL; *p* values indicated. *d.* day, *LT-HSCs* long-term HSCs, *ST-HSCs* short-term HSCs, *LSKs* Lin-Sca1+cKit+﻿, *MPPs* multipotent progenitor populations, *CMPs* common myeloid progenitors, *MEPs* megakaryocyte/erythroid progenitors, *GMPs* granulocyte/monocyte progenitors, *LBPs* lymphoid-biased progenitors, *CLPs* common lymphoid progenitors

Similar results were observed in C57BL/6 mice; however, the maximal effect on HSC populations occurred 2 days later than in the nude mice. Specifically, no differences were observed between the control and the ZOL-treated cohorts 3 days after administration, while at the 5-day time point, there was a 1.8-fold increase in HSCs (*p* = 0.04), a 2.0-fold increase in LT-HSCs (*p* = 0.03), and a 1.7-fold increase in ST-HSCs (*p* = 0.05), after which these numbers returned to baseline and remained stable until the experimental end point at day 20 (Fig. 1g, Table 1).

To determine whether increased cell proliferation could account for the observed changes in HSCs numbers, we injected ZOL-treated and vehicle-treated mice with BrdU and analyzed HSCs by flow cytometry. The BrdU+ HSCs as a percentage of total HSCs were not significantly different between the ZOL-treated and control-treated cohorts at any time point analyzed, even at time points as early as 24 and 48 hours after ZOL treatment (Fig. 1c, Additional file 5: Figure S3d). These data suggest that while ZOL treatment results in a significant expansion of HSCs in the marrow, the percentage of proliferating cells is constant and therefore, the increase in numbers of HSCs is likely due to differences in their proliferation rates rather than to HSCs amassing in a quiescence phase.

Given that the HSC numbers were significantly elevated at 3 days (nude mice) and 5 days (C57BL/6 mice) after ZOL treatment, we focused on these time points to determine if ZOL was increasing the numbers of functional HSCs. To do so, we performed CFU assays using BMCs isolated from both vehicle-treated and ZOL-treated mice at these time points. We found that ZOL significantly increased the number of CFUs in both strains of mice (nude mice, day 3: control 5.2 ± 1.4, ZOL 10.4 ± 1.6, *p* = 0.04; C57BL/6 mice, day 5: control 11.6 ± 4.0, ZOL 21.6 ± 5.7, *p* = 0.05) (Fig. 1d). Additionally, no increases in the number of CFUs were observed in the spleen or peripheral blood 3 days after ZOL administration as compared to control treatment, indicating that the effect of ZOL was specific to the bone marrow (Additional file 5: Figure S3c).

To determine whether the increased HSC function could be ascribed to direct effects of ZOL on HSCs, we isolated BMCs from naïve mice and subjected them to the CFU assay in the presence of either vehicle or 10 μM ZOL in vitro (a dose comparable to the 100 μg/kg dose used in vivo). Ten days after treatment, no differences in the number of CFUs between the groups were observed (Fig. 1e), suggesting that ZOL did not act directly on HSCs to mediate their expansion in vivo and that this expansion was not attributed to direct drug storage and release from the bone matrix.

We next determined whether ZOL affected the proliferation of other bone marrow hematopoietic cells. To start, we stained sections of tibiae with an antibody specific for the proliferation marker Ki67. The numbers of Ki67+ cells per field in the marrow of ZOL-treated nude mice were increased by around 82% (*p* < 0.0001) and in C57BL/6 mice by around 74% (*p* = 0.0007), compared to the respective vehicle controls (Fig. 1f). These results prompted us to carry out a more detailed analysis of the specific hematopoietic progenitor cell (HPC) populations at various time points after administration of control treatment or ZOL, using flow cytometric analysis (Additional file 1: Table S1, Additional file 2: Table S2 and Additional file 3: Figure S1).

Compared to control treatment, ZOL had significant effects on a number of BMC populations in the nude mice (Table 1, Fig. 1g). Specifically, there was a 1.3-fold increase in the Lin-Sca1+cKit+﻿ (LSK) populations (*p* = 0.02) accompanied by a non-significant 1.8-fold increase in multipotent progenitors (MPPs, LSK/CD150-/CD48+) 3 days after ZOL-treatment (*p* = 0.1). Common myeloid progenitor cell (CMP, Lin-Sca1-cKit+IL7Ra-CD34 + FcγRII/III-) numbers were elevated 2.0-fold (*p* = 0.03) and 3.4-fold (*p* = 0.01) at the 3-day and 5-day time points, respectively. Megakaryocyte-erythroid progenitors (MEPs, Lin-Sca1-cKit + IL7Ra-FCγRII/II-CD34-) were elevated 1.8-fold (*p* = 0.03) on day 5. Numbers of granulocyte-macrophage progenitors (GMPs, Lin-Sca1-cKit + IL7R-CD34 + FcγRII/III+) appeared to be elevated 5 days after ZOL treatment compared to the control cohort, although this change was not statistically significant (1.4-fold increase, *p* = 0.2). Common lymphoid progenitors (CLPs, Lin-/sca^int^/ckit^int^ILRa+), which are present in nude mice, were decreased 1.5-fold (*p* = 0.04) at the 5-day time point in the marrow of ZOL-treated mice. In all cases, the numbers of these various progenitor cells returned to baseline by day 10.

In the C57BL/6 mice, the changes in HPC numbers followed some of the same trends as those observed in the nude mice, although with different kinetics (Fig. 1g, Table 1). The numbers of MPPs were increased 1.4-fold by day 5 (*p* = 0.004) and 1.3-fold by day 10 (*p* < 0.0001). CMPs and MEPs were elevated 1.5-fold (*p* = 0.1) and 1.6-fold (*p* = 0.07), respectively, after 15 days, although these numbers were not statistically significant. GMPs were significantly elevated 1.3-fold (*p* = 0.02) at the 15-day time point in the marrow of ZOL-treated mice. In all cases, the numbers of these various progenitor cells returned to baseline by day 20. Additionally, we observed a significant 2.2-fold (*p* = 0.0003) decrease in the number lymphoid biased progenitor (LBP) populations in the C57BL/6 mice 5 days after ZOL treatment, after which these numbers were not statistically different from those in the control cohort. In these C57BL/6 mice, we also observed significant 3.1-fold (*p* = 0.0001) and 2.5-fold (*p* = 0.003) increases in the number of common lymphoid progenitor (CLP) populations 5 and 10 days after ZOL treatment, respectively. The decrease in LBPs and increase in CLPs indicate that it is possible that the effect of ZOL on the bone marrow drives LBPs toward differentiation into the downstream CLP populations.

Collectively, these results established that ZOL has profound and transient effects on hematopoiesis in both nude and C57BL/6 mice. In particular, the effects of ZOL appeared to favor differentiation toward common lymphoid progenitors and expansion of myeloid lineage progenitors, which may be accounted for by the significant expansion in HSCs. Additionally, our results suggest that the effects of ZOL on HSC proliferation and expansion are indirect.

### Effect of zoledronic acid on peripheral blood monocytes and neutrophils

A number of reports have demonstrated that bisphosphonate treatment reduces circulating monocytes and neutrophils [27, 28]. It is also well-established that hematopoiesis in the marrow compensates for significant alterations in circulating blood cell numbers [29]. Hence, we next investigated how the changes to hematopoietic cells in the marrow related to peripheral blood counts during the approximate 2-week time period after ZOL administration.

We analyzed circulating blood cell populations at various time points after ZOL or control treatment in both strains of mice. In the C57BL/6 mice, there was around 49% reduction in neutrophils (*p* = 0.05) and around 83% reduction in monocytes (*p* = 0.003) in the blood of the ZOL-treated cohort relative to the control cohort on day 5 (Fig. 1g, Table 2). Platelets were increased compared to control at 3, 5 and 10 days after ZOL administration, with approximately 49% elevation observed at the 5-day time point (*p* = 0.01) (Fig. 1g, Table 2). Moreover, on day 5 there were significant reductions in the numbers of eosinophils (*p* = 0.03) and basophils (*p* = 0.01) in the ZOL-treated mice (Additional file 5: Figure S3b).

In nude mice, we observed a trend toward reduced numbers of circulating neutrophils and monocytes with ZOL treatment relative to vehicle control, although these numbers were not statistically significant (Fig. 1g, Table 2). There were also no statistically significant differences in the numbers of other blood cell populations between cohorts at these time points in the nude mice (Table 2, Additional file 5: Figure S3a).

**Table 2:** ZOL-induced modifications to peripheral blood cell counts over time

		Control	Three days post ZOL	Five days post ZOL	Ten days post ZOL
Nude	Monocytes	0.37 ± 0.10	0.21 ± 0.09	0.27 ± 0.11	0.45 ± 0.11
	Neutrophils	2.78 ± 0.46	1.73 ± 0.48	1.86 ± 0.18	2.28 ± 0.65
C57BL/6	Monocytes	0.82 ± 0.20	0.28 ± 0.12*	0.14 ± 0.04*	0.62 ± 0.14
	Neutrophils	1.56 ± 0.16	0.98 ± 0.16*	0.79 ± 0.33*	1.41 ± 0.29
	Platelets	438.6 ± 32.10	477.0 ± 87.66	653.3 ± 68.50*	512.2 ± 64.50

Average numbers of indicated peripheral blood cells (K/μL ± SEM) from nude and C57BL/6 mice as determined by HEMAVET® at the indicated time points after control treatment or one 100-μg/kg dose of zoledronic acid (ZOL); *n* = 4–5 mice per group; *p ≤ 0.05

Taken together with the bone marrow analysis, these results indicate that reductions in peripheral neutrophils and monocytes were accompanied by an increase in their progenitor cells in the bone marrow after administration of a single, clinically relevant dose of ZOL. In the C57BL/6 mice, the increase in peripheral blood platelets observed 5 days after ZOL treatment preceded the increase in MEPs observed in the bone marrow, which was observed 15 days after ZOL treatment. Additionally, although we observed decreases in the LBP and increases in the CLP populations in the bone marrow of C57BL/6 mice 5 days after ZOL treatment, there was not a corresponding change in the number of lymphocytes in the peripheral blood observed at this same time point.

### Zoledronic acid induces changes to extracellular matrix and bone cell number and activity

As HSC maintenance and hematopoiesis are tightly regulated by cells in the endosteal niche, in particular osteoblasts and osteoclasts, we endeavored to determine whether the ZOL-induced changes in hematopoietic cell populations occurred in conjunction with the well-established effects on these particular bone cells. We therefore analyzed osteoclast and osteoblast parameters in the cohorts of nude mice at the time points at which we had analyzed hematopoietic stem cell populations.

**Fig. 2:**
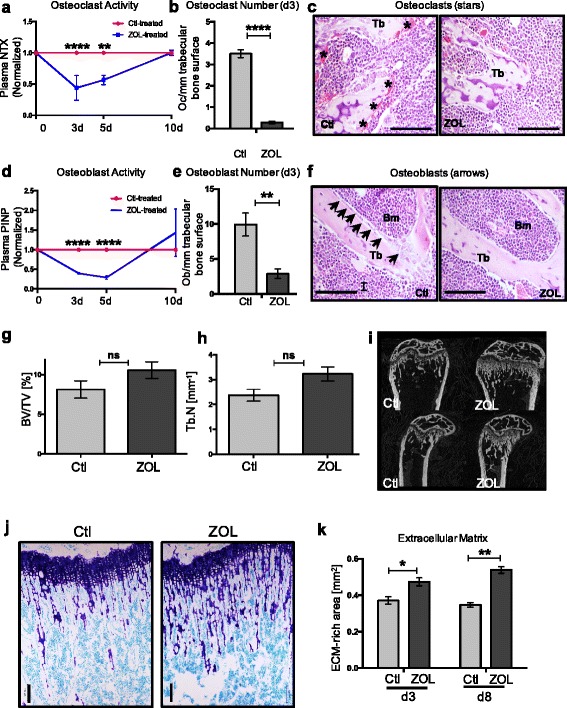
Effects of zoledronic acid (*ZOL*) on osteoclasts, osteoblasts and bone structure. **a** Relative osteoclast activity in nude mice determined by N-telopeptide of type 1 collagen (*NTX*) plasma levels at indicated time points after ZOL treatment (*n* = 4–5 per group); day (*d*) 3 ****p < 0.0001, **d5 *p* = 0.0005, d10 *p* > 0.999. **b** Osteoclast number/mm trabecular bone surface (*n* = 4 per group); ****p < 0.0001. **c** Representative sections of tartrate-resistant acid phosphatase-stained tibiae, with an H&E counterstain, 3 days after ZOL or control (*Ctl*) treatment in nude mice. Osteoclasts stain bright pink and are indicated by *asterisks*, *Tb* trabecular bone, *scale bar* 100 μm. **d** Relative osteoblast activity as measured by plasma procollagen I N-terminal propeptide (*PINP*) at indicated time points after ZOL treatment (*n* = 4–5/group); d3 ****p < 0.0001, d5 ****p < 0.0001, d10 *p* = 0.4. **e** Osteoblast number/mm trabecular bone surface (*n* = 4/group); **p = 0.007. **f** Representative H&E-stained section of tibiae from Ctl and ZOL-treated mice; *arrows* indicate osteoblasts, *Tb* trabecular bone, *Bm* bone marrow, *scale bar* 100 μm. Trabecular bone volume (bone volume/tissue volume, *BV/TV* in %) (**g**) and trabecular number (*Tb.N.* in mm^-1^) (**h**) of nude mouse femora 3 days after treatment (*n* = 3 for Ctl, n = 4 for ZOL). **i** Representative micro-computed CT cross-sections of right femora from Ctl or ZOL treated mice 3 days after treatment. **k** Average area (mm^2^) occupied by extracellular matrix in the metaphysis of nude mice at indicated time points after treatment (*n* = 3–4 mice/group); d3 *p = 0.02, d8 **p = 0.001. **j** Toluidine blue-stained sections of tibiae of nude mice 3 or 8 days after treatment with Ctl or one 100-ug/kg dose of ZOL. *Dark purple* stain shows bone proteoglycan (mastocytes, cartilage), *light blue* stain shows cell nuclei and calcified bone. *Scale bar* 100 μm

As expected, ZOL caused a significant reduction in osteoclast activity, as measured by N-telopeptide of type 1 collagen (NTX) plasma concentrations, at both 3 and 5 days after treatment, with levels returning to baseline by day 10 (day 3: *p* < 0.0001, day 5: *p* = 0.0005, day 10: *p* > 0.999; Fig. 2a). The decrease in osteoclast activity observed at day 3 was accompanied by an 11.7-fold reduction in the number of osteoclasts lining trabecular bone surfaces of the ZOL-treated cohort relative to the control-treated cohort (*p* < 0.0001; Fig. 2b, c).

Although the long-term therapeutic effects of bisphosphonates are thought to be a consequence of direct inhibition of osteoclast-mediated bone resorption, there is increasing evidence that these drugs also affect osteoblasts, due to the tight coupling of osteoclast and osteoblast activity [30, 31]. We observed a transient but significant reduction in plasma procollagen I N-terminal propeptide (PINP) (a measure of osteoblast activity) 3 and 5 days after ZOL administration, after which it was not significantly different relative to the control cohort (day 3: *p* < 0.0001, day 5: *p* < 0.0001, day 10: *p* = 0.4, Fig. 2d). Concomitant with these findings, the number of osteoblasts lining trabecular bone surfaces was reduced 3.4-fold relative to control (*p* = 0.007, Fig. 2e, f).

To establish whether these changes in bone cell number and activity manifested in alterations to bone structure, we analyzed trabecular bone volume 3 days after ZOL administration. As expected, no significant alterations in trabecular bone volume or number were observed at this early time point (Fig. 2g-i). However, as measured by proteoglycan staining, we observed increased extracellular matrix deposition in the metaphysis 3 days after treatment (1.3-fold increase relative to control, *p* = 0.02) with a further increase 8 days after ZOL treatment (1.5-fold increase relative to control, *p* = 0.001, Fig. 2j, k). Although gross total bone density had not yet been measurably affected at these relatively early time points, increases in extracellular matrix deposition in the metaphysis in this study was consistent with our previous finding that ZOL increases trabecular bone extracellular matrix deposition and that increases in bone volume are not observed until 10 days following treatment [21].

These results established that a single dose of ZOL decreases the number and activity of osteoblasts and osteoclasts within a few days of administration, after which activity returns to baseline. Moreover, although gross changes in bone density were not apparent, significant changes to the bone extracellular matrix had begun to manifest. The kinetics of these changes to bone mirrored those with which alterations to hematopoietic progenitors (described above) had occurred in the wake of ZOL treatment.

### Effects of zoledronic acid on cells in the vascular niche

Proliferative HSCs are reported to reside in peri-vascular niches within the bone [32, 33]. Moreover, it has been suggested that ZOL has anti-angiogenic activity in vitro and reduces circulating pro-angiogenic vascular endothelial growth factor (VEGF) in patients with cancer [34–37]. Based on these findings, and on a recent report that HSC maintenance is regulated by specific bone marrow blood vessel types [23], we investigated the effects of ZOL on the bone microvasculature. To do so, we stained bone tissue sections from ZOL and control treated mice for the endothelial cell marker endomucin, which is expressed by the vessels in both the metaphysis and diaphysis [23].

**Fig. 3:**
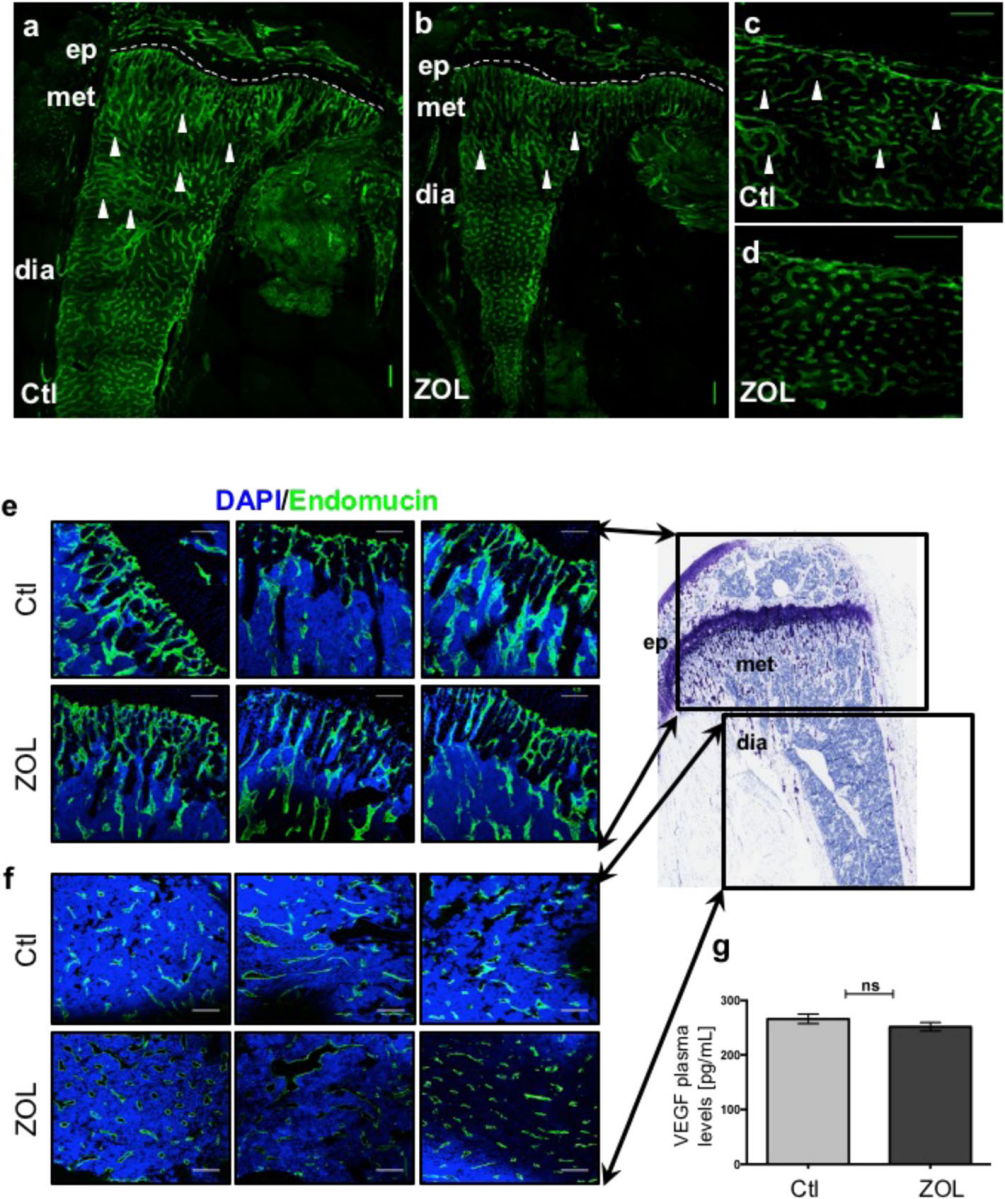
Effects of a single dose of zoledronic acid (*ZOL*) on bone marrow vasculature. **a**-**d** Bone marrow vasculature was visualized 3 days after control (*Ctl*) or ZOL treatment using immunofluorescence staining against the vascular endothelial cell marker endomucin (*green*) on 30-μm-thick sections of gelatin-embedded tibiae: 8 × 8 tile scans captured with × 20 objective using the Nikon Eclipse Ti, NIS-Elements-software Version 4.30, CFI Plan Fluor 20x MI (NA 0.75); *scale bars* 250 μm, *arrows* indicate branched vasculature, *dashed lines* represent growth plate demarcation, *Ep* epiphysis, *Met* metaphysis, *Dia* diaphysis. **e**-**f** Z-stacks with a depth of 20 μm of the metaphyseal vasculature (**e**) and confocal images of the diaphyseal bone marrow vasculature (**f**), acquired with the Nikon A1 confocal microscope, NIS-Elements-software Version 4.30, CFI Plan Fluor 20x MI (NA 0.75), Endomucin-positive vascular endothelial cells (*green*; Alexa555), nuclei (*blue*; 4',6-diamidino-2-phenylindole (*DAPI*)); *scale bars* 100 μm; *n* = 3/group. Phase-contrast image (*right*) is a toluidine blue stain of a nude mouse tibia to indicate the regions of imaging for the vasculature stains. **g** Average mouse plasma vascular endothelial growth factor (*VEGF*) concentration (pg/mL) 3 days after a single dose of ZOL or Ctl treatment (*n* = 4 for Ctl, *n* = 3 for ZOL). *ns* not significant

In the control cohort, we observed a distinct organization of endomucin-positive microvasculature, with elongated, branched tubular vessels occupying the metaphysis and a more sinusoidal patterning in the diaphysis, as expected. Although no quantitative difference was observed in the number of vessels and average length of vessels (Additional file 6: Figure S4b), the endomucin-positive vasculature of ZOL-treated animals appeared to comprise shorter, discontinuous vessels with a deficit of sinusoidal patterning in the diaphysis and an apparent elongation of the branched zone within the metaphysis (Fig. 3a-f, Additional file 6: Figure S4a).

Using a second endothelial cell-surface marker, CD31, which is expressed by the vessels in the metaphysis but is absent in the diaphysis [23], we observed no overt differences in organization and structure of the CD31^+^ bone marrow vasculature (Additional file 6: Figure S4a) in the ZOL-treated cohort when compared to control; however, the zone occupied by columnar vessels appeared to be extended, in agreement with the endomucin staining (Fig. 3e). VEGF plasma levels were not affected 3 days after a single dose of ZOL or control (*p* = 0.3, control: 266.2 ± 8.80 pg/mL vs ZOL: 251.7 ± 7.92 pg/mL, Fig. 3g), indicating that ZOL did not change VEGF plasma levels at this time point.

Although not easily quantifiable, our observations support the notion that a single dose of ZOL modifies the organization of the bone microvasculature in areas known to comprise HSC niches and thus could, at least in part, account for the expansion in HSCs that we observed 3 days after ZOL administration. Whether these ZOL-induced modulations to the microvasculature have significant effects on hematopoiesis will be the subject of future studies.

### Effect of zoledronic acid-treated bone marrow cells on breast tumor outgrowth

In recent clinical trials, adjuvant ZOL has been correlated with reduced breast cancer recurrence, although the precise mechanism(s) of action for this protective effect remains unknown [25]. Given that hematopoietic cells play an important role in breast cancer progression [38, 39] and that our findings demonstrated that ZOL has profound effects on hematopoietic stem and progenitor cells, we wished to determine whether the ZOL-induced alterations in hematopoiesis have an effect on breast tumor outgrowth. We based our modeling on the notion that the outgrowth of disseminated cancer cells encountering a bone marrow environment that had been modified by ZOL could be profoundly affected by those ZOL-induced changes to hematopoietic cells that we had observed.

**Fig. 4:**
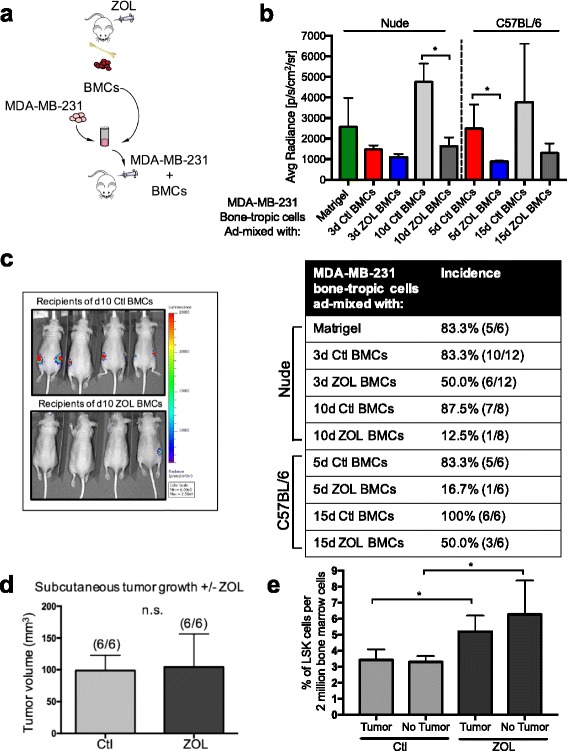
Zoledronic acid (*ZOL*) generates tumor-suppressive bone marrow cells. **a** Experimental design to test bone marrow tumor support function. Bone marrow cells (*BMCs*) were harvested from control (*Ctl*)-treated or ZOL-treated donor mice 3 and 10 days (nude) or 5 and 15 days (C57Bl/6) after treatment (*n* = 3 donor mice/cohort): 7.5 × 10^5^ BMCs were directly admixed with 2.5 × 10^5^ luciferase + MDA-MB-231 BO2F11 bone tropic human breast cancer cells and admixtures immediately injected subcutaneously into cohorts of recipient nude mice (*n* = 3–6 recipient mice per cohort with bilateral subcutaneous injections; **b** and **c** are representative images of one of three biological replications). Tumor growth was monitored over a 14-day experimental time course. **b** Average radiance signal at each injection site per cohort at experimental endpoint (day (d)14) resulting from admixtures of MDA-MB-231 BO2F11 tumor cells with BMCs from indicated Ctl-treated or ZOL-treated donor nude mice; *p < 0.05. *Table* indicates incidence of tumor formation resulting from admixtures of breast tumor cells with BMCs from indicated donor mice. **c** Representative In vivo imaging system images of luciferase + MDA-MB-231 BO2F11 tumors resulting from admixtures with BMCs from Ctl-treated or ZOL-treated donor nude mice (10 days after treatment of donor mice). Imaging was acquired at the 14-day experimental end point. **d** Nude mice were treated with 100 μL of vehicle or 100 μg/kg ZOL; 3 days later, mice were injected subcutaneously with 2.5 × 10^5^ MDA-MB-231 BO2F11 and tumor growth was monitored over a 14-day time period. Average tumor volume (mm^3^) for indicated cohorts is shown; *n.s.* not statistically significant. **e** Nude mice were injected orthotopically with MDA-MB-231 bone-tropic cells and tumors grew for 30 days before mice were given one dose of Ctl treatment or ZOL (100 μg/kg). BMCs were harvested 3 days after Ctl or ZOL administration and Lin-Sca1+cKit+﻿(*LSKs*) were analyzed by flow cytometry, *p < 0.05

To exclusively test the effects of ZOL-modified bone marrow in the absence of overt bone disease, we compared the ability of BMCs isolated from control-treated and ZOL-treated animals to modulate tumor cell growth using our previously reported BMC functional assay [40] (Fig. 4a). To do so, BMCs were isolated from mice at selected time points after control or ZOL treatment and admixed with MDA-MB-231 bone-tropic (BO2F11) human breast tumor cells in a ratio of 3:1. The admixtures were immediately injected subcutaneously into recipient cohorts of nude mice and tumor incidence and progression were assessed over a period of 14 days (Fig. 4a). In all cases, we confirmed that the vast majority of cells in the BMC preparations were CD45+ hematopoietic cells, with <0.005–0.02% of the cells being CD45-negative (data not shown).

We first started with BMCs harvested at those time points at which maximal effects on hematopoiesis were observed, namely, 3 days in nude mice and 5 days in C57BL/6 mice. BMCs prepared from the control cohort of nude donor mice 3 days after vehicle treatment had no effect on tumor growth; the incidence of tumor formation was approximately 83% in the cohort of mice that had received these admixtures and in a cohort of mice that had been injected with tumor cells only (i.e., without any admixed BMCs) (Fig. 4b, c). In striking contrast, BMCs prepared from nude mice 3 days after ZOL treatment significantly inhibited tumor outgrowth; only 50% of the injected admixtures formed tumors (Fig. 4b). Similarly, tumor incidence was only approximately 17% in the mice that had received donor BMCs harvested from C57BL/6 mice 5 days after ZOL treatment while tumors formed in approximately 83% of the mice that had received control BMCs (Fig. 4b). These results established that ZOL generates tumor-suppressive bone marrow but did not reveal whether the changes we had observed in hematopoiesis were correlated with the inhibitory function of the bone marrow.

We reasoned that if ZOL-induced changes to hematopoiesis could define the tumor-inhibitory bone marrow, then BMCs isolated from control-treated and ZOL-treated mice at time points when HSC and HPC counts had returned to baseline (by 10 days in nude mice and 15 days in C57BL/6 mice) should no longer have tumor suppressive capacity. Interestingly, however, BMCs isolated from ZOL-treated donor mice at these later time points after treatment also had tumor-inhibitory function. Specifically, BMCs harvested from nude donor mice 10 days after ZOL treatment inhibited the outgrowth of MDA-MB-231 bone-tropic tumor cells (approximately 12% incidence) as compared to control-treated donor bone marrow (approximately 88% incidence) (Fig. 4b, c). Likewise, donor BMCs harvested from C57BL/6 mice 15 days after ZOL treatment resulted in decreased incidence of tumor formation (approximately 50%) relative to the control-treated donor cohort (100%) (Fig. 4b). Furthermore, in those tumors that did form, 5-day ZOL-treated BMCs in the nude mice reduced tumor growth kinetics compared to control-treated bone marrow cells (Additional file 7: Figure S5a) and 10 day ZOL-treated BMCs in the nude mice and 5 day ZOL-treated BMCs in the C57BL/6 mice reduced tumor size compared to control-treated bone marrow cells (Fig. 4b and Additional file 7: Figure S5b).

To rule out the possibility that tumor growth inhibition was the consequence of some residual drug that may have been retained in the donor bone marrow and introduced during the admixing process, we tested whether the tumor cells were susceptible to ZOL treatment. First, MDA-MB-231 BO2F11 cells were treated in vitro with a dose comparable to the dose used in vivo and no statistically significant differences were observed in the ZOL-treated cells compared to the control-treated cells in the cell proliferation growth assays (Additional file 7: Figure S5c). Second, to determine the likelihood that ZOL affected tumor cells in vivo, mice were administered one dose of ZOL 3 days prior to subcutaneous injection with MDA-MB-231 bone-tropic cells, and then re-dosed with ZOL every 5 days. Tumor growth was monitored over 14 days. There were no statistically significant differences in total tumor burden at the experimental end point in the ZOL-treated cohorts compared to the control cohorts (Fig. 4d), indicating that ZOL does not directly inhibit growth of these tumor cells, consistent with earlier reports [41].

As mammary tumors may also impact hematopoiesis, we wished to determine if the impact of ZOL on hematopoiesis occurred in the presence of a primary mammary tumor. We injected MDA-MB-231 bone-tropic cells orthotopically and after 30 days of tumor growth, we treated the mice with control or ZOL for 3 days before assessing the bone marrow by flow cytometry. Consistent with our earlier observations (Fig. 1) we observed expansion of the LSK compartment with ZOL, even in the presence of a primary mammary tumor (Fig. 4e).

Taken together, our findings established that ZOL treatment generates tumor-suppressive bone marrow cells. Moreover, these findings indicated that the tumor-suppressive action of ZOL is longer-lasting than the transient alterations to bone and hematopoietic cell numbers.

## Discussion

The bisphosphonate ZOL is increasingly being investigated in the adjuvant and neoadjuvant settings for reducing breast cancer recurrence in bone [2, 42–46]. A number of theories have been proposed to explain the protective effect of ZOL against breast cancer recurrence [47–49]. Our novel results provide an alternative explanation — that administration of a single, clinically relevant dose of ZOL is sufficient to generate bone marrow cells capable of directly suppressing breast cancer tumor growth.

Our findings have implications for breast tumors that metastasize to bone by suggesting that ZOL renders the bone marrow inhospitable for disseminated tumor cells. Indeed, in a clinical study of patients with breast cancer, ZOL significantly reduced disseminated tumor cells in the bone marrow [50]. Given that bone marrow-derived cells (BMDCs) also play a critical role in supporting primary tumors and extra-skeletal metastases [4], our results serve as a prerequisite for understanding how BMDCs within the tumor microenvironment might be affected by adjuvant ZOL. This theory is supported by certain preclinical studies reporting that ZOL decreases recruitment of BMDCs, such as tumor-infiltrating immune cells and tumor-associated macrophages, to peripheral tumor sites [34, 35, 51].

Previous reports from our laboratory have repeatedly demonstrated that gene expression changes that result in secretion of various cytokines, chemokines and growth factors, and not changes in BMC numbers, confer tumor-modulating activities to bone marrow cells [52, 53]. Likewise, published qPCR array studies of nude mouse BMCs a week after treatment demonstrate that ZOL reduces expression of a range of genes, including known regulators of the endosteal stem cell niches and of hematopoietic and vascular progenitor cell mobilization [54]. Such changes in gene expression could help to define ZOL-induced tumor-suppressive BMCs. Hence, future studies will be directed toward understanding gene expression signatures associated with ZOL-treated BMCs and determining which of these changes render the BMCs tumor-suppressive.

## Conclusions

Our results indicate that the ability of a single, clinically relevant dose of ZOL to generate tumor-suppressive bone marrow persists longer than its effects on hematopoiesis. In a clinical study of patients with osteoporosis, one dose of ZOL induced statistically significant increases in bone mineral density and decreases in bone resorption as late as 36 months after treatment [55]. Results such as these lend support to the notion that a less frequent dosing regimen may sustain the clinical benefits of ZOL while reducing the potential for the toxicity (specifically, osteonecrosis of the jaw) that has been associated with frequent ZOL treatment [55]. Moreover, pre-clinical studies such as ours suggest possibilities for capitalizing on the beneficial effects of ZOL on reducing breast cancer metastasis in the bone, identification of bone marrow cell biomarkers that predict response to ZOL and improving responses to existing therapies.

## Additional files

Additional file 1: Table S1. Flow cytometry cell-surface markers used to quantify indicated hematopoietic stem and progenitor cell populations. (PDF 16 kb)
Additional file 2: Table S2. Flow cytometry antibody dilutions and product information. (PDF 16 kb)
Additional file 3: Figure S1. Flow cytometry gating strategies employed for identification and quantification of hematopoietic stem and progenitor populations in the bone marrow. (PDF 552 kb)
Additional file 4: Figure S2. Schematic illustration of osteoblast, osteoclast and proteoglycan quantifications. **a** Parameters for osteoclast and osteoblast identification. Osteoblast and osteoclast number on all trabecular bone surfaces was scored 200 μm away from the growth plate. **b** Parameters for proteoglycan-rich matrix quantification. (PDF 1542 kb)
Additional file 5: Figure S3. Peripheral blood counts represented as average fold change in nude (**a**) and C57BL/6 (**b**) mice 3 and 5 days after ZOL treatment, respectively (*n* = 4 − 5/group). **c** CFU assay of bone marrow, peripheral blood and spleen 3 days after CTL or ZOL treatment; *n* = 5 nude mice/cohort, **p* < 0.05. **d** BrDU-positive HSCs as a percentage of total HSCs per nude mouse femur at early time points post ZOL treatment; *n.s.* not significant; *n* = 5 mice/cohort, 1 femur per mouse. (PDF 986 kb)
Additional file 6: Figure S4. Effects of a single dose of zoledronic acid (*ZOL*) on CD31+ bone marrow vasculature. Treatment effects of ZOL or control (*Ctl*) on CD31-positive bone marrow vasculature in the metaphysis 3 days after ZOL treatment in the nude mice (**a**) was visualized using immunofluorescence staining against the vascular endothelial cell marker CD31 on 30-μm-thick sections of gelatin-embedded tibiae. Z-stacks with a depth of 20 μm were acquired using the Nikon A1 confocal microscope, NIS-Elements-software Version 4.30, CFI Plan Fluor 20x MI (NA 0.75), *yellow* CD31-positive vascular endothelial cells (Alexa555), *blue* nuclei (DAPI), *n* = 3/group. **b** Endomucin-positive vessels (*red*) were tracked manually using Aperio ImageScope. Number of vessels and their average length are shown, *n* = 3/group. (PDF 3075 kb)
Additional file 7: Figure S5.
**a** Growth kinetics of tumors after injecting host nude mice subcutaneously with admixtures of MDA-MB-231 BO2F11 cells with either Matrigel control (*green*) BMCs from nude mice 5 days after CTL treatment (*red*) or BMCs from nude mice 5 days after ZOL treatment (*blue*); *n* = 12 host mice per cohort, one subcutaneous tumor injection per mouse. Bone marrow obtained from three donor mice per cohort. **b** Average radiance signal of palpable tumors that grew at each injection site per cohort at experimental endpoint (day (*d*)14) resulting from admixtures of MDA-MB-231 BO2F11 tumor cells with BMCs from indicated CTL-treated or ZOL-treated donor nude mice. **c** MDA-MB-231 BO2F11 cells were treated in vitro with indicated doses of ZOL: 3 days later cell toxicity was measured using the CytoTox-Glo Assay. *Red arrow* indicates ZOL dose that is comparable to in vivo dose (based on estimate of mouse blood volume as 8% of total mouse body weight). (PDF 458 kb)

## Abbreviations

AEC: 3-Amino-9-ethylcarbazole
BMCs: Bone marrow cells
BMDCs: Bone marrow derived cells
BrdU: Bromodeoxyuridine
CFU: Colony forming unit
CLP: Common lymphoid progenitors
CMP: Common myeloid progenitors
DMEM: Dulbecco’s modified Eagle’s medium
EDTA: Ethylenediaminetetraacetic acid
ELISA: Enzyme-linked immunosorbent assay
FBS: Fetal bovine serum
FFPE: Formalin-fixed paraffin-embedded
GMP: Granulocyte-macrophage progenitors
H&E: Hematoxylin and eosin
HBBS: Hank’s Balanced Buffer Solution
HPC: Hematopoietic progenitor cells
HSC: Hematopoietic stem cell
i.p.: Intraperitoneal
IVIS: In vivo imaging system
LBP: Lymphoid biased progenitor
LSK: Lin-Sca1+cKit+
LT-HSC: Long-term hematopoietic stem cell
MEP: Megakaryocyte-erythroid progenitors
MPP: Multipotent progenitors
NTX: N-telopeptide of type 1 collagen
PBS: Phosphate-buffered saline
PFA: Paraformaldehyde
PINP: Procollagen I N-terminal propeptide
PVP: Polyvinylpyrrolidone
qPCR: Quantitative polymerase chain reaction
RBC: Red blood cell
SEM: Standard error of the mean
ST-HSC: Short-term hematopoietic stem cell
STR: Short tandem repeat
TRAP: Tartrate-resistant acid phosphatase
VEGF: Vascular endothelial growth factor
VOI: Volume of interest
ZOL: Zoledronic acid
μCT: Micro-computed tomography

## References

[1] Coleman RE (2006). Clinical features of metastatic bone disease and risk of skeletal morbidity. Clin Cancer Res.

[2] Coleman R, Powles T, Paterson A, Gnant M, Anderson S, Diel I, Gralow J, von Minckwitz G, Moebus V, Bergh J (2015). Adjuvant bisphosphonate treatment in early breast cancer: meta-analyses of individual patient data from randomised trials. Lancet.

[3] Gnant M, Mlineritsch B, Luschin-Ebengreuth G, Kainberger F, Kassmann H, Piswanger-Solkner JC, Seifert M, Ploner F, Menzel C, Dubsky P (2008). Adjuvant endocrine therapy plus zoledronic acid in premenopausal women with early-stage breast cancer: 5-year follow-up of the ABCSG-12 bone-mineral density substudy. Lancet Oncol.

[4] McAllister SS, Weinberg RA (2014). The tumour-induced systemic environment as a critical regulator of cancer progression and metastasis. Nat Cell Biol.

[5] Warr MR, Pietras EM, Passegue E (2011). Mechanisms controlling hematopoietic stem cell functions during normal hematopoiesis and hematological malignancies. Wiley Interdiscip Rev Syst Biol Med.

[6] Orkin SH, Zon LI (2008). Hematopoiesis: an evolving paradigm for stem cell biology. Cell.

[7] Morrison SJ, Scadden DT (2014). The bone marrow niche for haematopoietic stem cells. Nature.

[8] Xie Y, Yin T, Wiegraebe W, He XC, Miller D, Stark D, Perko K, Alexander R, Schwartz J, Grindley JC (2009). Detection of functional haematopoietic stem cell niche using real-time imaging. Nature.

[9] Wang H, Yu C, Gao X, Welte T, Muscarella AM, Tian L, Zhao H, Zhao Z, Du S, Tao J (2015). The osteogenic niche promotes early-stage bone colonization of disseminated breast cancer cells. Cancer Cell.

[10] Calvi LM, Adams GB, Weibrecht KW, Weber JM, Olson DP, Knight MC, Martin RP, Schipani E, Divieti P, Bringhurst FR (2003). Osteoblastic cells regulate the haematopoietic stem cell niche. Nature.

[11] Kollet O, Dar A, Shivtiel S, Kalinkovich A, Lapid K, Sztainberg Y, Tesio M, Samstein RM, Goichberg P, Spiegel A (2006). Osteoclasts degrade endosteal components and promote mobilization of hematopoietic progenitor cells. Nat Med.

[12] Cho KA, Joo SY, Han HS, Ryu KH, Woo SY (2010). Osteoclast activation by receptor activator of NF-kappaB ligand enhances the mobilization of hematopoietic progenitor cells from the bone marrow in acute injury. Int J Mol Med.

[13] Zhang J, Niu C, Ye L, Huang H, He X, Tong WG, Ross J, Haug J, Johnson T, Feng JQ (2003). Identification of the haematopoietic stem cell niche and control of the niche size. Nature.

[14] Mendez-Ferrer S, Michurina TV, Ferraro F, Mazloom AR, Macarthur BD, Lira SA, Scadden DT, Ma'ayan A, Enikolopov GN, Frenette PS (2010). Mesenchymal and haematopoietic stem cells form a unique bone marrow niche. Nature.

[15] Rodan GA (1998). Mechanisms of action of bisphosphonates. Annu Rev Pharmacol Toxicol..

[16] Rogers MJ, Chilton KM, Coxon FP, Lawry J, Smith MO, Suri S, Russell RG (1996). Bisphosphonates induce apoptosis in mouse macrophage-like cells in vitro by a nitric oxide-independent mechanism. J Bone Miner Res.

[17] Rogers MJ, Frith JC, Luckman SP, Coxon FP, Benford HL, Monkkonen J, Auriola S, Chilton KM, Russell RG (1999). Molecular mechanisms of action of bisphosphonates. Bone.

[18] Jain N, Weinstein RS (2009). Giant osteoclasts after long-term bisphosphonate therapy: diagnostic challenges. Nat Rev Rheumatol.

[19] Fournier PG, Stresing V, Ebetino FH, Clezardin P (2010). How do bisphosphonates inhibit bone metastasis in vivo?. Neoplasia.

[20] Rogers MJ, Crockett JC, Coxon FP, Monkkonen J (2011). Biochemical and molecular mechanisms of action of bisphosphonates. Bone.

[21] Haider MT, Holen I, Dear TN, Hunter K, Brown HK (2014). Modifying the osteoblastic niche with zoledronic acid in vivo-potential implications for breast cancer bone metastasis. Bone..

[22] Soki FN, Li X, Berry J, Koh A, Sinder BP, Qian X, Kozloff KM, Taichman RS, McCauley LK (2013). The effects of zoledronic acid in the bone and vasculature support of hematopoietic stem cell niches. J Cell Biochem.

[23] Kusumbe AP, Ramasamy SK, Adams RH (2014). Coupling of angiogenesis and osteogenesis by a specific vessel subtype in bone. Nature.

[24] Wetterwald A, van der Pluijm G, Que I, Sijmons B, Buijs J, Karperien M, Lowik CW, Gautschi E, Thalmann GN, Cecchini MG (2002). Optical imaging of cancer metastasis to bone marrow: a mouse model of minimal residual disease. Am J Pathol.

[25] Coleman RE, Gregory W, Marshall H, Wilson C, Holen I (2013). The metastatic microenvironment of breast cancer: clinical implications. Breast..

[26] Jilka RL (2013). The relevance of mouse models for investigating age-related bone loss in humans. J Gerontol A Biol Sci Med Sci.

[27] Kuiper JW, Forster C, Sun C, Peel S, Glogauer M (2012). Zoledronate and pamidronate depress neutrophil functions and survival in mice. Br J Pharmacol.

[28] Kalyan S, Chandrasekaran V, Quabius ES, Lindhorst TK, Kabelitz D (2014). Neutrophil uptake of nitrogen-bisphosphonates leads to the suppression of human peripheral blood gamma delta T cells. Cell Mol Life Sci.

[29] Mendelson A, Frenette PS (2014). Hematopoietic stem cell niche maintenance during homeostasis and regeneration. Nat Med.

[30] Viereck V, Emons G, Lauck V, Frosch KH, Blaschke S, Grundker C, Hofbauer LC (2002). Bisphosphonates pamidronate and zoledronic acid stimulate osteoprotegerin production by primary human osteoblasts. Biochem Biophys Res Commun.

[31] von Knoch F, Jaquiery C, Kowalsky M, Schaeren S, Alabre C, Martin I, Rubash HE, Shanbhag AS (2005). Effects of bisphosphonates on proliferation and osteoblast differentiation of human bone marrow stromal cells. Biomaterials.

[32] Kiel MJ, Yilmaz OH, Iwashita T, Yilmaz OH, Terhorst C, Morrison SJ (2005). SLAM family receptors distinguish hematopoietic stem and progenitor cells and reveal endothelial niches for stem cells. Cell.

[33] Taichman RS (2005). Blood and bone: two tissues whose fates are intertwined to create the hematopoietic stem-cell niche. Blood.

[34] Coscia M, Quaglino E, Iezzi M, Curcio C, Pantaleoni F, Riganti C, Holen I, Monkkonen H, Boccadoro M, Forni G (2010). Zoledronic acid repolarizes tumour-associated macrophages and inhibits mammary carcinogenesis by targeting the mevalonate pathway. J Cell Mol Med.

[35] Melani C, Sangaletti S, Barazzetta FM, Werb Z, Colombo MP (2007). Amino-biphosphonate-mediated MMP-9 inhibition breaks the tumor-bone marrow axis responsible for myeloid-derived suppressor cell expansion and macrophage infiltration in tumor stroma. Cancer Res.

[36] Allegra A, Oteri G, Nastro E, Alonci A, Bellomo G, Del Fabro V, Quartarone E, Alati C, De Ponte FS, Cicciu D (2007). Patients with bisphosphonates-associated osteonecrosis of the jaw have reduced circulating endothelial cells. Hematol Oncol.

[37] Santini D, Vincenzi B, Galluzzo S, Battistoni F, Rocci L, Venditti O, Schiavon G, Angeletti S, Uzzalli F, Caraglia M (2007). Repeated intermittent low-dose therapy with zoledronic acid induces an early, sustained, and long-lasting decrease of peripheral vascular endothelial growth factor levels in cancer patients. Clin Cancer Res.

[38] Joyce JA, Pollard JW (2009). Microenvironmental regulation of metastasis. Nat Rev Cancer.

[39] Gao D, Mittal V (2009). The role of bone-marrow-derived cells in tumor growth, metastasis initiation and progression. Trends Mol Med.

[40] McAllister SS, Gifford AM, Greiner AL, Kelleher SP, Saelzler MP, Ince TA, Reinhardt F, Harris LN, Hylander BL, Repasky EA (2008). Systemic endocrine instigation of indolent tumor growth requires osteopontin. Cell.

[41] Ottewell PD, Woodward JK, Lefley DV, Evans CA, Coleman RE, Holen I (2009). Anticancer mechanisms of doxorubicin and zoledronic acid in breast cancer tumor growth in bone. Mol Cancer Ther.

[42] Kroep JR, Charehbili A, Coleman RE, Aft RL, Hasegawa Y, Winter MC, Weilbaecher K, Akazawa K, Hinsley S, Putter H (2016). Effects of neoadjuvant chemotherapy with or without zoledronic acid on pathological response: a meta-analysis of randomised trials. Eur J Cancer..

[43] Gnant M, Mlineritsch B, Stoeger H, Luschin-Ebengreuth G, Heck D, Menzel C, Jakesz R, Seifert M, Hubalek M, Pristauz G (2011). Adjuvant endocrine therapy plus zoledronic acid in premenopausal women with early-stage breast cancer: 62-month follow-up from the ABCSG-12 randomised trial. Lancet Oncol.

[44] Coleman RE, Marshall H, Cameron D, Dodwell D, Burkinshaw R, Keane M, Gil M, Houston SJ, Grieve RJ, Barrett-Lee PJ (2011). Breast-cancer adjuvant therapy with zoledronic acid. N Engl J Med.

[45] Coleman R, de Boer R, Eidtmann H, Llombart A, Davidson N, Neven P, von Minckwitz G, Sleeboom HP, Forbes J, Barrios C (2013). Zoledronic acid (zoledronate) for postmenopausal women with early breast cancer receiving adjuvant letrozole (ZO-FAST study): final 60-month results. Ann Oncol.

[46] Hadji P, Coleman R, Gnant M, Green J (2012). The impact of menopause on bone, zoledronic acid, and implications for breast cancer growth and metastasis. Ann Oncol.

[47] Aft R, Perez JR, Raje N, Hirsh V, Saad F (2012). Could targeting bone delay cancer progression? Potential mechanisms of action of bisphosphonates. Crit Rev Oncol Hematol.

[48] Winter MC, Holen I, Coleman RE (2008). Exploring the anti-tumour activity of bisphosphonates in early breast cancer. Cancer Treat Rev.

[49] Young RJ, Coleman RE (2013). Zoledronic acid to prevent and treat cancer metastasis: new prospects for an old drug. Future Oncol.

[50] Aft R, Naughton M, Trinkaus K, Watson M, Ylagan L, Chavez-MacGregor M, Zhai J, Kuo S, Shannon W, Diemer K (2010). Effect of zoledronic acid on disseminated tumour cells in women with locally advanced breast cancer: an open label, randomised, phase 2 trial. Lancet Oncol.

[51] Junankar S, Shay G, Jurczyluk J, Ali N, Down J, Pocock N, Parker A, Nguyen A, Sun S, Kashemirov B (2015). Real-time intravital imaging establishes tumor-associated macrophages as the extraskeletal target of bisphosphonate action in cancer. Cancer Discov.

[52] Elkabets M, Gifford AM, Scheel C, Nilsson B, Reinhardt F, Bray MA, Carpenter AE, Jirstrom K, Magnusson K, Ebert BL (2011). Human tumors instigate granulin-expressing hematopoietic cells that promote malignancy by activating stromal fibroblasts in mice. J Clin Invest.

[53] Marsh T, Wong I, Sceneay J, Barakat A, Qin Y, Sjodin A, Alspach E, Nilsson B, Stewart SA, McAllister SS (2016). Hematopoietic age at onset of triple-negative breast cancer dictates disease aggressiveness and progression. Cancer Res.

[54] Ottewell PD, Wang N, Brown HK, Reeves KJ, Fowles CA, Croucher PI, Eaton CL, Holen I (2014). Zoledronic acid has differential antitumor activity in the pre- and postmenopausal bone microenvironment in vivo. Clin Cancer Res.

[55] Brown JE, Ellis SP, Lester JE, Gutcher S, Khanna T, Purohit OP, McCloskey E, Coleman RE (2007). Prolonged efficacy of a single dose of the bisphosphonate zoledronic acid. Clin Cancer Res.

