# Supplementary material for: Zoledronic acid alters hematopoiesis and generates breast tumor-suppressive bone marrow cells
Source: Breast Cancer Res. 2017 Mar 6;19:23. doi: 10.1186/s13058-017-0815-8 (PMC5339994; doi:10.1186/s13058-017-0815-8)
Supplement: Additional file 1: Table S1. — Flow cytometry cell-surface markers used to quantify indicated hematopoietic stem and progenitor cell populations. (PDF 16 kb) [file 13058_2017_815_MOESM1_ESM.pdf]

HSC/HPC flow markers	
LSK Population	Lin-sca1+ckit- (LSK)
Hematopoietic Stem Cell (HSC)	LSK CD150+CD48+CD34-Flt3-
Long-term HSC (LT-HSC)	LSK CD34- Flt3-
Short-term HSC (ST-HSC)	LSK CD34- Flt3+
Multipotent progenitor (MPP)	LSK CD150-CD48+
Lymphoid Biased Progenitor (LBP)	LSK CD150-CD48-
Common Myeloid Progenitor (CMP)	Lin-sca1-ckit+IL7Ra-CD34+FCγRII/III-
Granulocyte-monocyte progenitor (GMP)	Lin-sca1-ckit+IL7Ra-CD34+FCγRII/III+
Common lymphoid progenitor (CLP)	Lin-sca1^{int}ckit^{int}IL7Ra+
Megakaryocyte/Erythroid Progenitor (MEP)	Lin-sca1-ckit+IL7Ra-CD34-FCγRII/III-
