# Supplementary material for: Zoledronic acid alters hematopoiesis and generates breast tumor-suppressive bone marrow cells
Source: Breast Cancer Res. 2017 Mar 6;19:23. doi: 10.1186/s13058-017-0815-8 (PMC5339994; doi:10.1186/s13058-017-0815-8)
Supplement: Additional file 2: Table S2. — Flow cytometry antibody dilutions and product information. (PDF 16 kb) [file 13058_2017_815_MOESM2_ESM.pdf]

Antibody	Alternate Names	Flurophore	Clone	Company	Catalog No.	Dilution
Sca1	Ly6A-E	BV605	D7	BioLegend	108133	1 to 100
cKit	CD117	BV786	2B8	BD	564012	1 to 200
CD150	SLAM	AF488	TC15	BioLegend	115815	1 to 100
CD48	BCM1	APC Cy7	HM48-1	BioLegend	103431	1 to 100
CD34		AF488		BioLegend		1 to 100
IL7Ra	CD127	PE Cy7	A7R34	BioLegend	135013	1 to 400
FcγRII/III	CD16/32	PE	93	BioLegend	101307	1 to 400
Flt3	CD135	PE	A2F10	BioLegend	135305	1 to 40
Hematopoietic Cocktail	lineage	PB		BioLegend	133310	1 to 10
BrDU		APC		BD	552598	1 to 200
CD45		APC Cy7		eBioscience	47045182	1 to 200
CD3		PE		BD	553240	1 to 100
