# Supplementary figures and images for: Zoledronic acid alters hematopoiesis and generates breast tumor-suppressive bone marrow cells
Source: Breast Cancer Res. 2017 Mar 6;19:23. doi: 10.1186/s13058-017-0815-8 (PMC5339994; doi:10.1186/s13058-017-0815-8)

## Lymphocytes

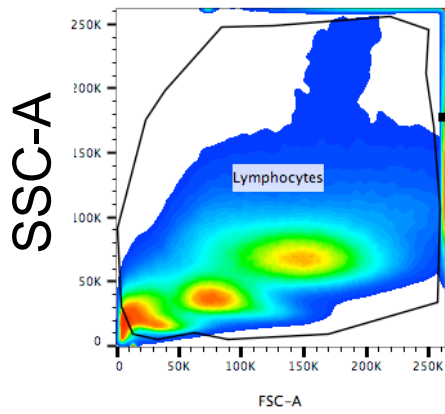

## Single Cells

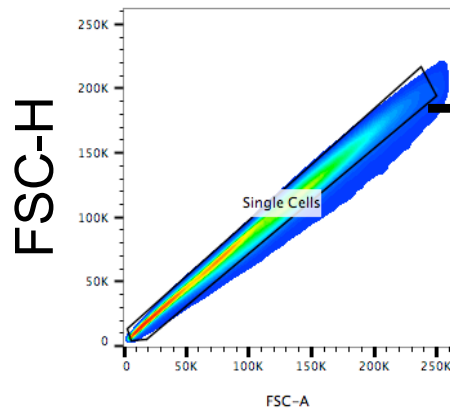

## Live/Dead

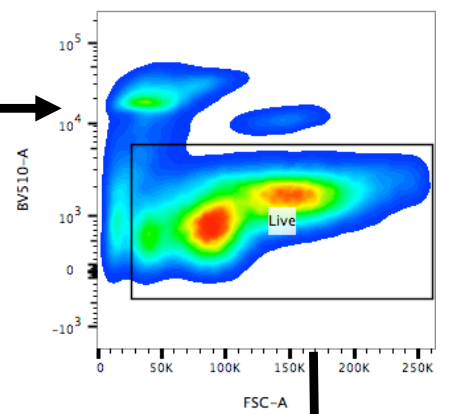

## Sca1

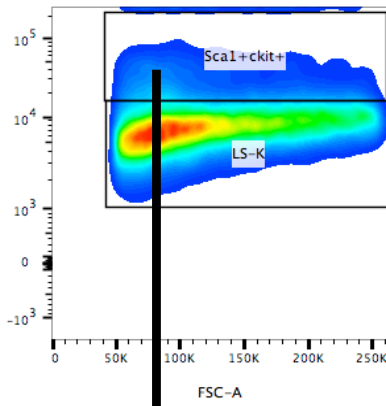

## ckit

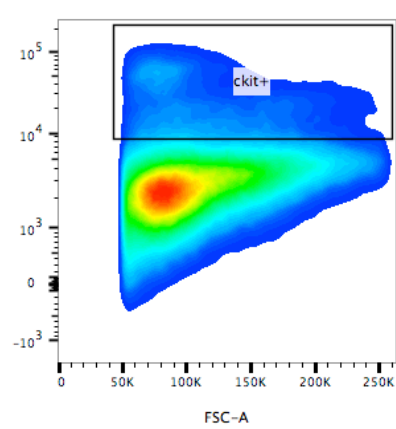

## Lineage

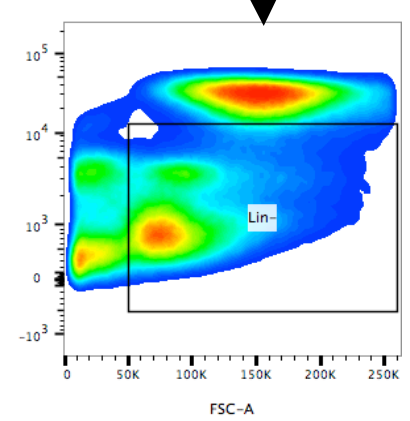

## CD34

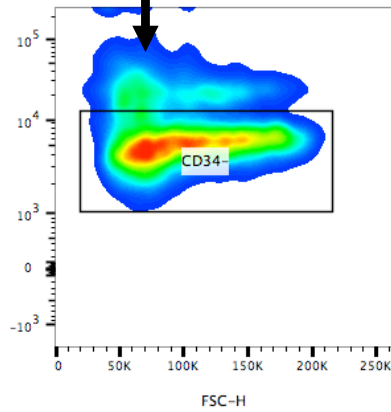

## Flt3

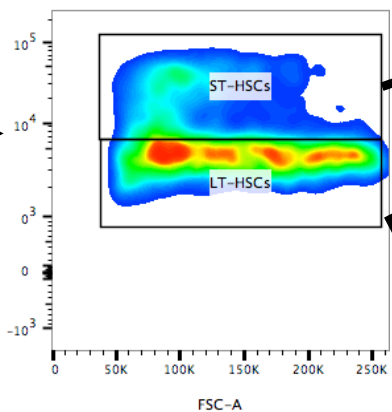

## BrDU

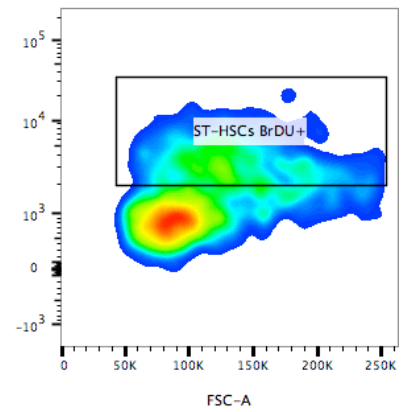

## BrDU

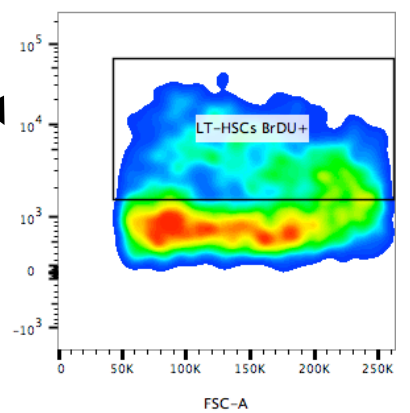

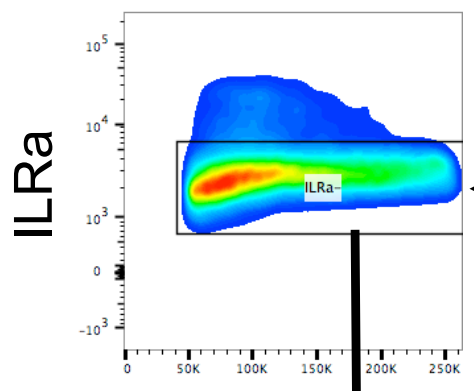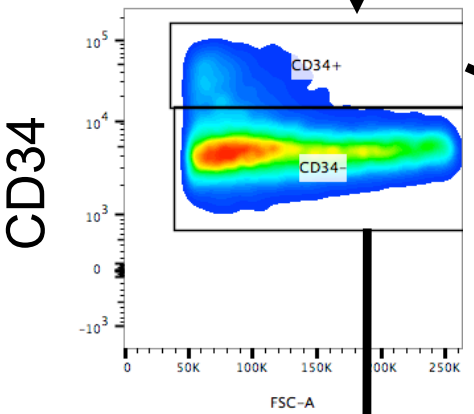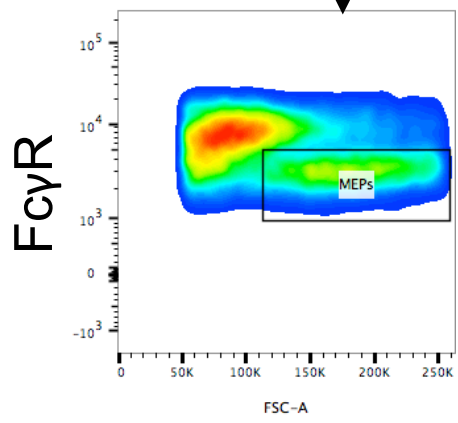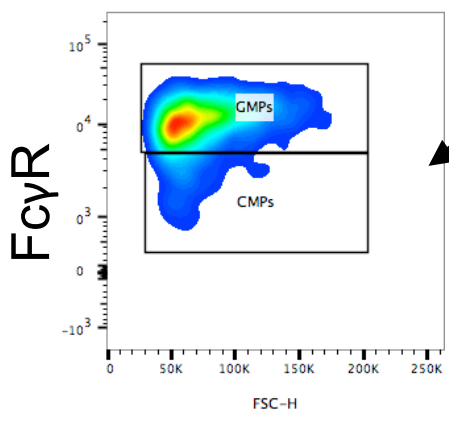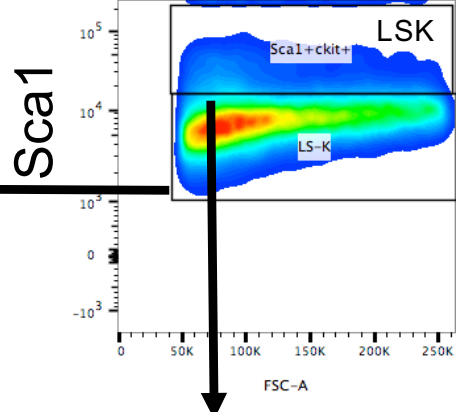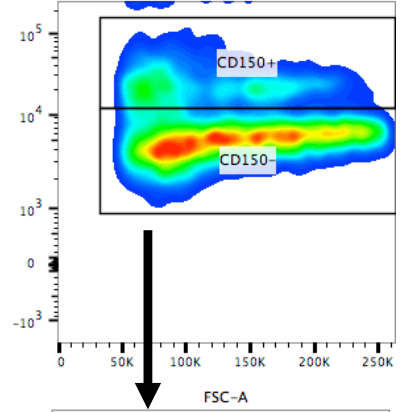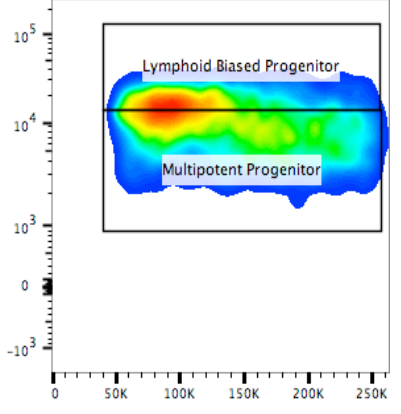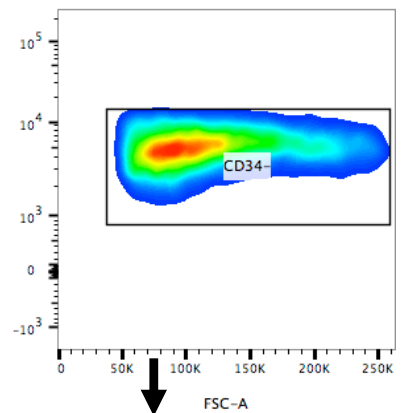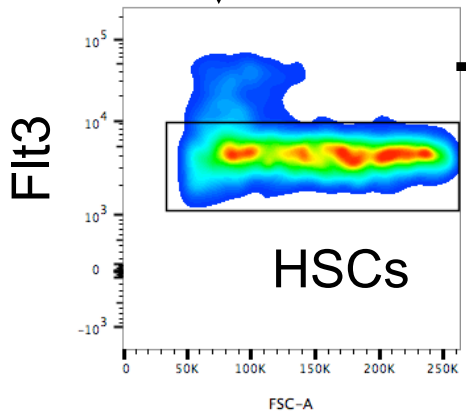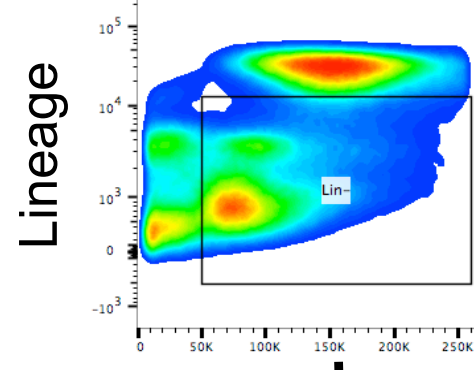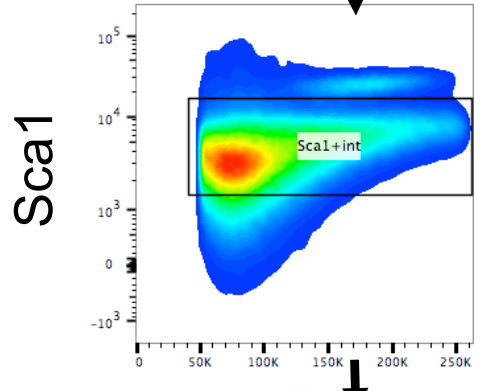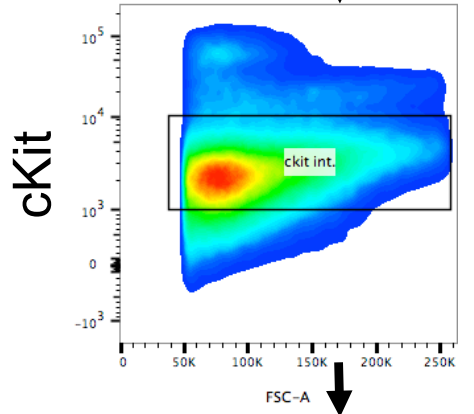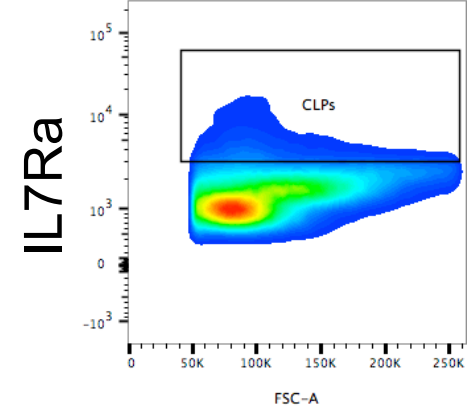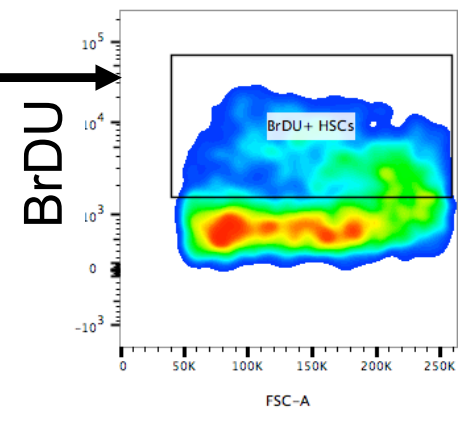

Supplement: Additional file 3: Figure S1. — Flow cytometry gating strategies employed for identification and quantification of hematopoietic stem and progenitor populations in the bone marrow. (PDF 552 kb) [file 13058_2017_815_MOESM3_ESM.pdf]

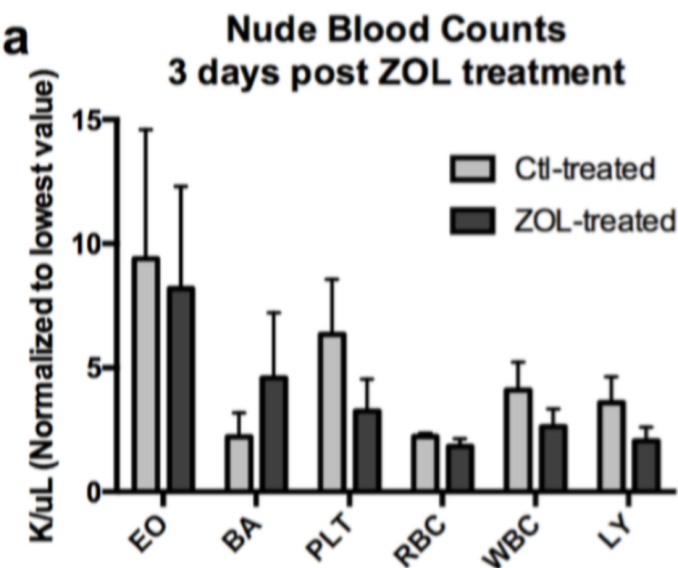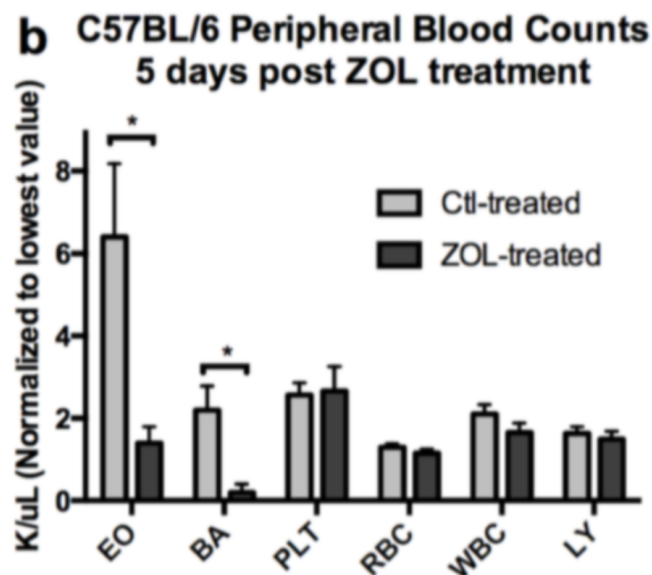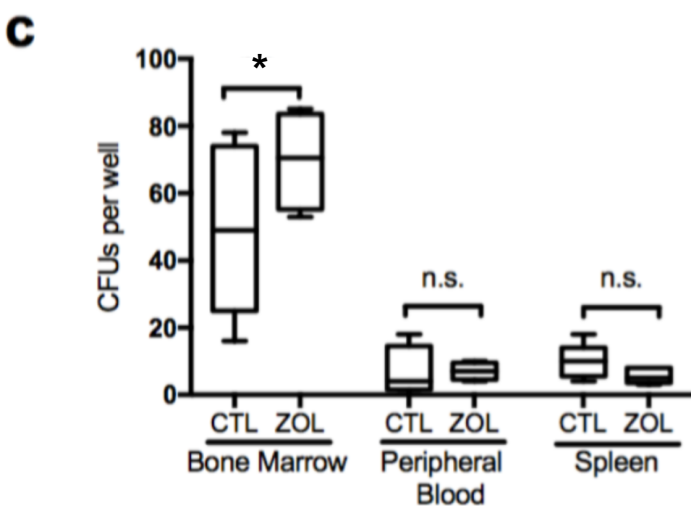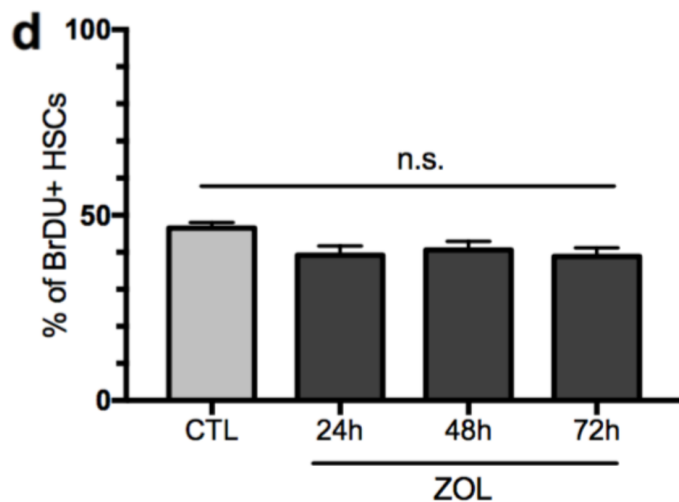

Supplement: Additional file 5: Figure S3. — Peripheral blood counts represented as average fold change in nude (a) and C57BL/6 (b) mice 3 and 5 days after ZOL treatment, respectively (n = 4 − 5/group). c CFU assay of bone marrow, peripheral blood and spleen 3 days after CTL or ZOL treatment; n = 5 nude mice/cohort, *p < 0.05. d BrDU-positive HSCs as a percentage of total HSCs per nude mouse femur at early time points post ZOL treatment; n.s. not significant; n = 5 mice/cohort, 1 femur per mouse. (PDF 986 kb) [file 13058_2017_815_MOESM5_ESM.pdf]

**a**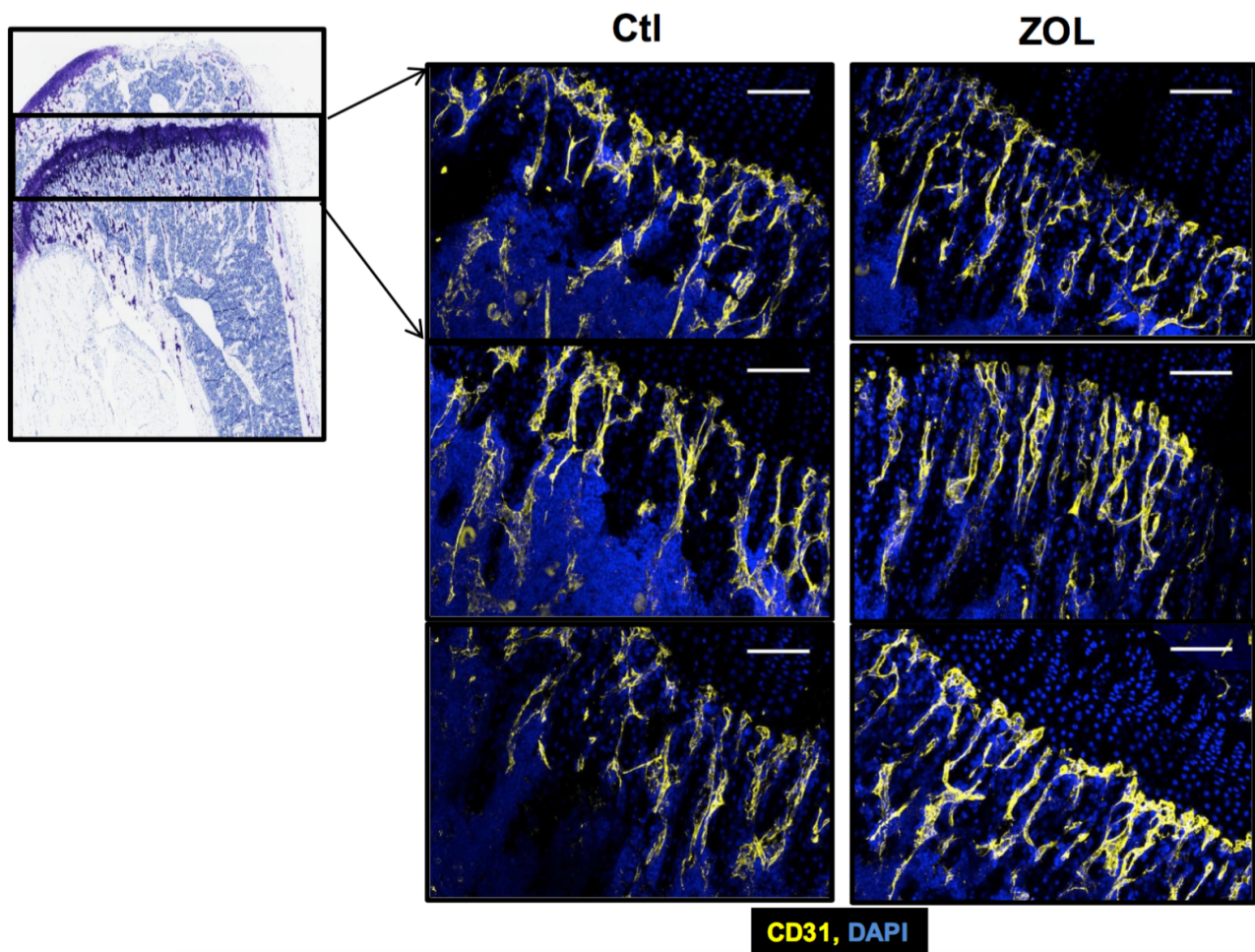**b**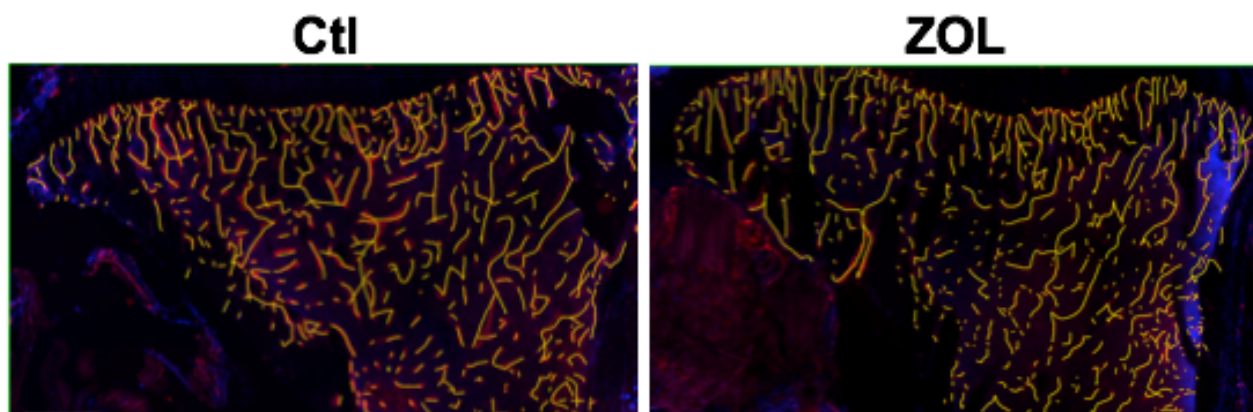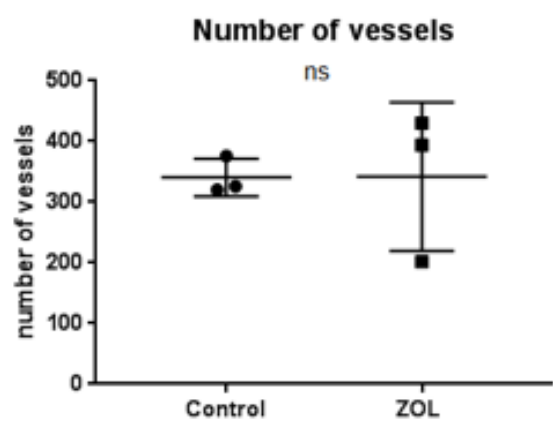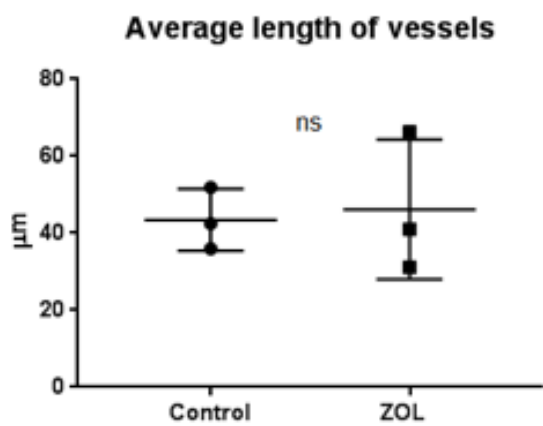

Supplement: Additional file 6: Figure S4. — Effects of a single dose of zoledronic acid (ZOL) on CD31+ bone marrow vasculature. Treatment effects of ZOL or control (Ctl) on CD31-positive bone marrow vasculature in the metaphysis 3 days after ZOL treatment in the nude mice (a) was visualized using immunofluorescence staining against the vascular endothelial cell marker CD31 on 30-μm-thick sections of gelatin-embedded tibiae. Z-stacks with a depth of 20 μm were acquired using the Nikon A1 confocal microscope, NIS-Elements-software Version 4.30, CFI Plan Fluor 20x MI (NA 0.75), yellow CD31-positive vascular endothelial cells (Alexa555), blue nuclei (DAPI), n = 3/group. b Endomucin-positive vessels (red) were tracked manually using Aperio ImageScope. Number of vessels and their average length are shown, n = 3/group. (PDF 3075 kb) [file 13058_2017_815_MOESM6_ESM.pdf]

**a**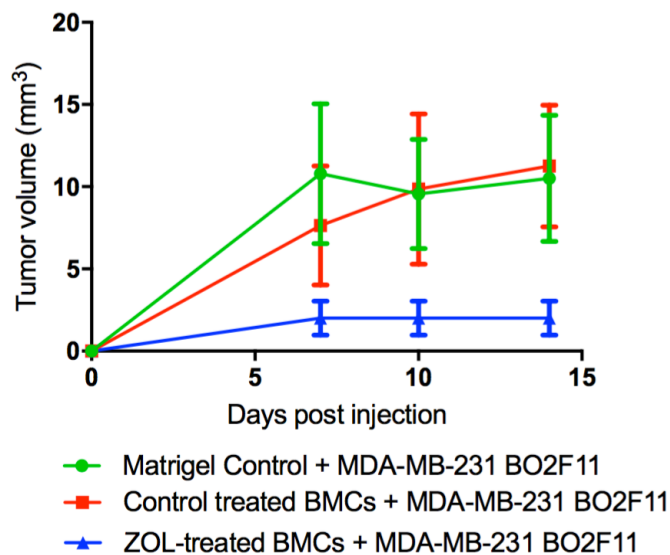**b**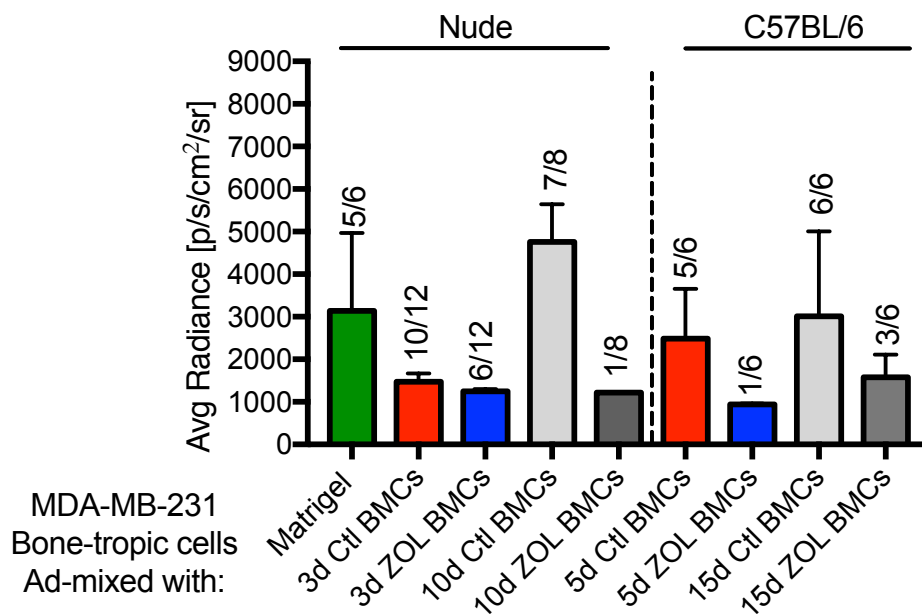**c**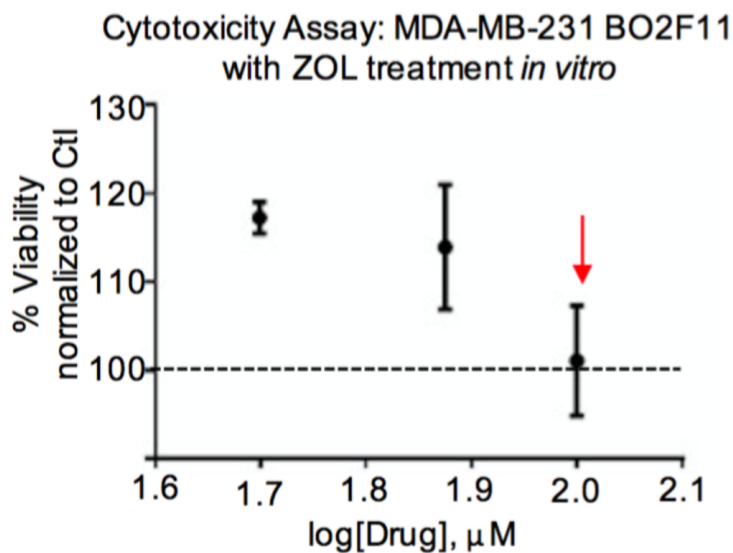

Supplement: Additional file 7: Figure S5. — a Growth kinetics of tumors after injecting host nude mice subcutaneously with admixtures of MDA-MB-231 BO2F11 cells with either Matrigel control (green) BMCs from nude mice 5 days after CTL treatment (red) or BMCs from nude mice 5 days after ZOL treatment (blue); n = 12 host mice per cohort, one subcutaneous tumor injection per mouse. Bone marrow obtained from three donor mice per cohort. b Average radiance signal of palpable tumors that grew at each injection site per cohort at experimental endpoint (day (d)14) resulting from admixtures of MDA-MB-231 BO2F11 tumor cells with BMCs from indicated CTL-treated or ZOL-treated donor nude mice. c MDA-MB-231 BO2F11 cells were treated in vitro with indicated doses of ZOL: 3 days later cell toxicity was measured using the CytoTox-Glo Assay. Red arrow indicates ZOL dose that is comparable to in vivo dose (based on estimate of mouse blood volume as 8% of total mouse body weight). (PDF 458 kb) [file 13058_2017_815_MOESM7_ESM.pdf]
