# Supplementary material for: Zoledronic acid alters hematopoiesis and generates breast tumor-suppressive bone marrow cells
Source: Breast Cancer Res. 2017 Mar 6;19:23. doi: 10.1186/s13058-017-0815-8 (PMC5339994; doi:10.1186/s13058-017-0815-8)

a Osteoblast and osteoclast quantification

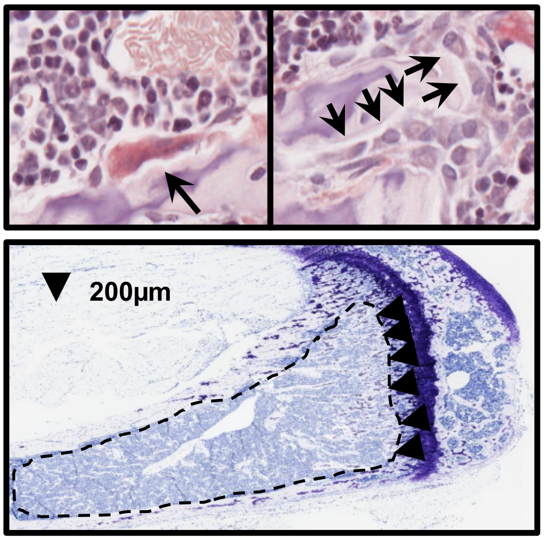

Osteoclast	Osteoblast
Multiple nuclei	Single, distinct nucleus
Ruffled border	Large golgi complex
In contact with bone surface	Appear in groups, in contact with bone surface
Pink, TRAP positive	Cuboidal shape

b Quantification of proteoglycan-rich matrix

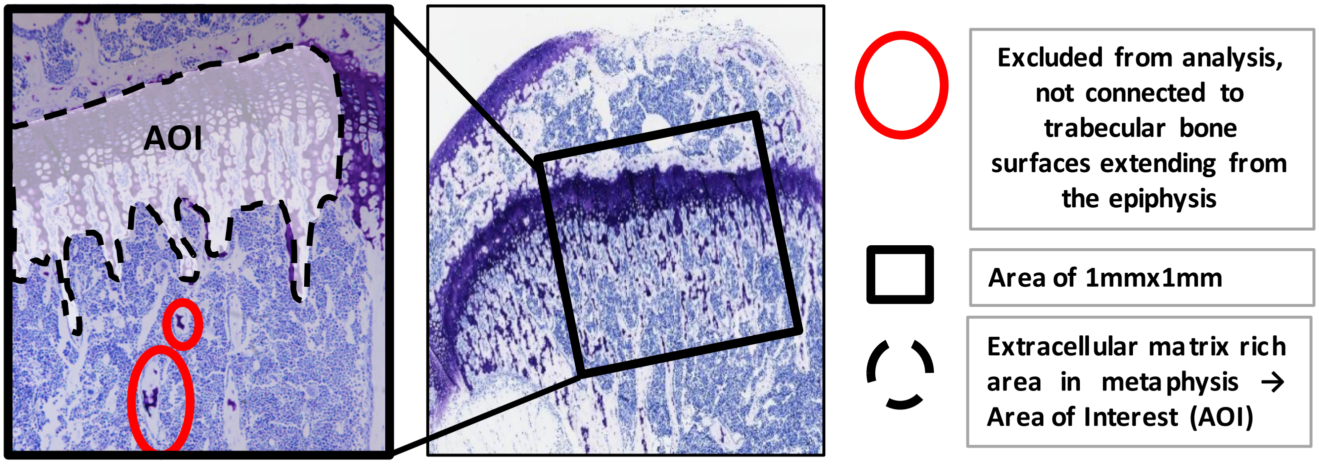

Supplement: Additional file 4: Figure S2. — Schematic illustration of osteoblast, osteoclast and proteoglycan quantifications. a Parameters for osteoclast and osteoblast identification. Osteoblast and osteoclast number on all trabecular bone surfaces was scored 200 μm away from the growth plate. b Parameters for proteoglycan-rich matrix quantification. (PDF 1542 kb) [file 13058_2017_815_MOESM4_ESM.pdf]
